# An overview of pink eye infection to evaluate its medications: group decision-making approach with 2-tuple linguistic *T*-spherical fuzzy WASPAS method

**DOI:** 10.3389/frai.2024.1496689

**Published:** 2025-01-21

**Authors:** M. Waheed Rasheed, Hind Y. Saleh, Areen A. Salih, Jahangeer Karamat, Muhammad Bilal

**Affiliations:** ^1^Department of Mathematics, COMSATS University Islamabad, Vehari, Pakistan; ^2^Department of Mathematics, College of Basic Education, University of Duhok, Duhok, Iraq; ^3^Department of Mathematics, College of Computer Science and Mathematics, University of Mosul, Mosul, Iraq; ^4^Department of Mathematics, Division of Science and Technology, University of Education, Lahore, Pakistan

**Keywords:** 2TL*T*-SFS, Schweizer-Sklar weighted average operator, Schweizer-Sklar weighted geometric operator, WASPAS, medication for pink eye infection

## Abstract

An infectious eye illness known as pink eye results in ocular redness, irritation, and mucus. Schools are an especially vulnerable region for dissemination because they can propagate that contagious disease quickly via direct or indirect interactions. Choosing the right medication to treat pink eye infection is typically thought of as an intricate multi-attribute group decision-making concern. The goal of this research is to construct a multi-attribute group decision-making framework that assesses six pink eye treatment medications, including Bleph-10, Moxeza, Zymar, Romycin, Polytrim, and Bacticin. The constructed multi-attribute group decision-making framework includes the following scenario: (1) In contrast to other types of fuzzy sets, the 2-tuple linguistic *T*-spherical fuzzy set (2TL*T*-SFS) looks to be a potent tool for dealing with informational inconsistencies in decision-making scenarios; (2) in order to render the 2TL*T*-SF accumulation details processing more flexible, the addition, multiplication, scalar multiplication, and exponential laws that are predicated on the Schweizer-Sklar collection of *t*-conorms and *t*-norms are described; (3) the Schweizer-Sklar weighted average and Schweizer-Sklar weighted geometric operators are then put forward employing the aforementioned operations to combine the data; (4) subsequently, using newly developed operators (referred to as 2TL*T*-SF Schweizer-Sklar weighted average and 2TL*T*-SF Schweizer-Sklar weighted geometric), this work enhances the conventional weighted aggregated sum product assessment (WASPAS) approach. The computation procedure for this methodology is thoroughly given to rank the alternatives; (5) to confirm the viability of the suggested approach, thorough computational and simulation assessments are conducted. An examination of the developed and existing research is compared to demonstrate the benefits of the suggested analysis.

## 1 Introduction

As a consequence of inflammation, the transparent membrane that covers the lid of the eye and the lens of the eye turns pink, giving the appearance of red eyes. This membrane is referred to by its medical term as the conjunctiva. When the eyes are bloated and irritated, the minuscule vessels of blood that are found in the conjunctiva become more noticeable. As a consequence of this, the whites of the eyes have a pinkish or reddish hue to them. Pink eye is also known as conjunctivitis in some circles. Pink eye is almost always caused by an infection that is viral. Viruses are the most common cause. Likewise, it could be caused by a fungal infection, an inflammatory response, or, in the case of newborns, a weeping channel that is only somewhat accessible. Even though pink eye can be a pain, it rarely impairs eyesight. Pink eye irritation may be lessened with some treatments. Since pink eye can spread quickly, getting a quick diagnosis and following the appropriate precautions can help contain its spread. Pink eyes can sometimes heal by themselves. The need to see a doctor is, nevertheless, rare. It is strongly advised that even though your child will be agitated, he or she not rub the infected eye because doing so can make it redder. A virus, bacteria, or allergy can all be to blame for pink eyes. It might be brought on by a certain object, dust, or even pollen. If the person played in a chlorinated pool, a response to the chlorine may have caused the red eye. A person can contract this virus from another person. Your child may contract it, for example, by touching an infected person or using that person's tissue. Wearing the sick person's sunglasses or eyeglasses might also spread the disease. Viruses, germs, allergies, or other irritants are possible causes of this illness. Redness, watery eyes, and a thin discharge are just a few of the signs of viral conjunctivitis, which is frequently linked to respiratory illnesses.

A thicker discharge and more severe symptoms characterize bacterial conjunctivitis, which is brought on by bacteria like Staphylococcus aureus. Itching and allergic conjunctivitis are not communicable and are brought on by allergens like pollen. Similar inflammatory reactions can also be brought on by irritants like smoke or chemicals. Redness, tearing, discharge, itching, sensitivity to light, and swelling are among the symptoms. An extensive eye exam is required for diagnosis, and the specific method of therapy will be determined by the primary cause. While bacterial conjunctivitis may need antibiotic therapy, viral conjunctivitis frequently goes away on its own. Avoiding allergens is how allergic conjunctivitis is treated, and in irritant situations, the irritant needs to be removed. Maintaining proper cleanliness, refraining from touching the eyes, and getting medical help when required are crucial for controlling and halting the spread of pink eye. A disorder known as conjunctivitis, also known more colloquially as pink eye, is characterized by warmth and blisters of the membranes that line the interior of the eye. Pink eye can be caused by a number of different things, including sensitivities, fungal germs, malware, and toxic irritants. Occasionally it is a manifestation of a malady that lasts for a long period of duration. In the majority of instances, pink eye is caused by germs with either malware or bacterium.

There are several potential causes of pink eye, including:

- Viruses, such as adenoviruses (common cold viruses), can cause viral conjunctivitis. The condition has an elevated risk of contagiousness and can be transmitted via explicit or oblique exposure to the ocular release of an individual who is sick.
- Pathogenic conjunctivitis frequently arises from microbial pathogens such as Staphylococcus aureus or Streptococcus influenza. Moreover, it possesses an elevated rate of transmissibility and often necessitates the administration of antiseptic eye drops or lotion for therapeutic intervention.
- As soon as the conjunctiva interacts with contaminants such as spores, dusting bug dander, fur from pets, or particular compounds, a condition known as inflammatory conjunctivitis can develop. It is not communicable and can be treated with corticosteroid eye drops or drugs that can be taken orally.
- Chemical irritants, such as smoke, smog, chlorine in swimming pools, or foreign bodies, can cause non-infectious conjunctivitis. Avoiding the irritant and using artificial tears may help alleviate symptoms.

The following are some of the potential signs of pink eye:

- The presence of erythema in the sclera or palpebral conjunctiva.
- Itchy or irritated eyes.
- Excessive tearing from the eye.
- Swollen eyelids.
- Crusty eyelashes, especially in the morning.
- Sensitivity to light.

**Figure 1:**
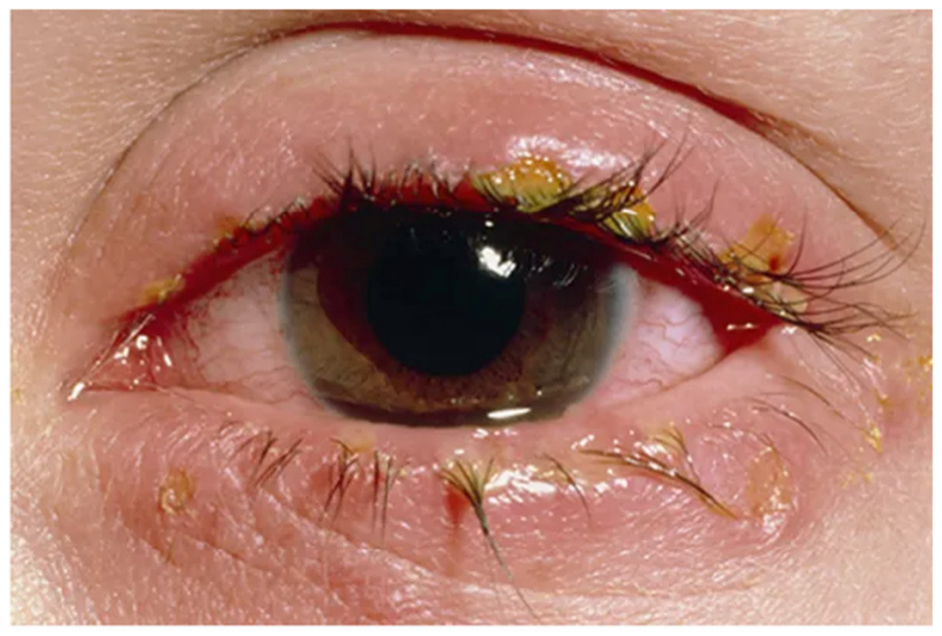
Crusty eyelids. Reproduced from WebMD, LLC, https://www.webmd.com/eye-health/eye-health-conjunctivitis#1-4.

**Figure 2:**
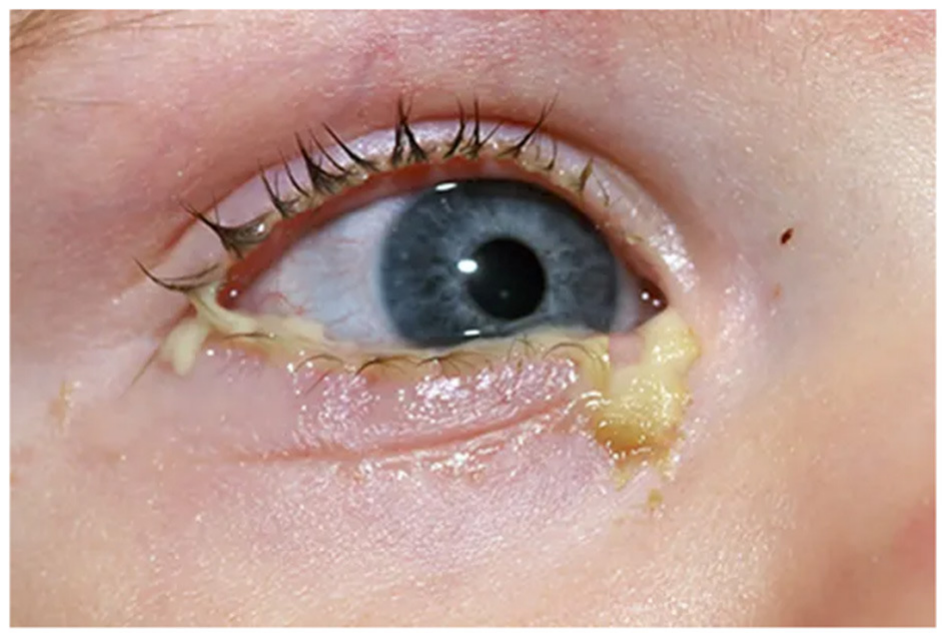
Drainage from the eyes. Reproduced from WebMD, LLC, https://www.webmd.com/eye-health/eye-health-conjunctivitis#1-4.

**Figure 3:**
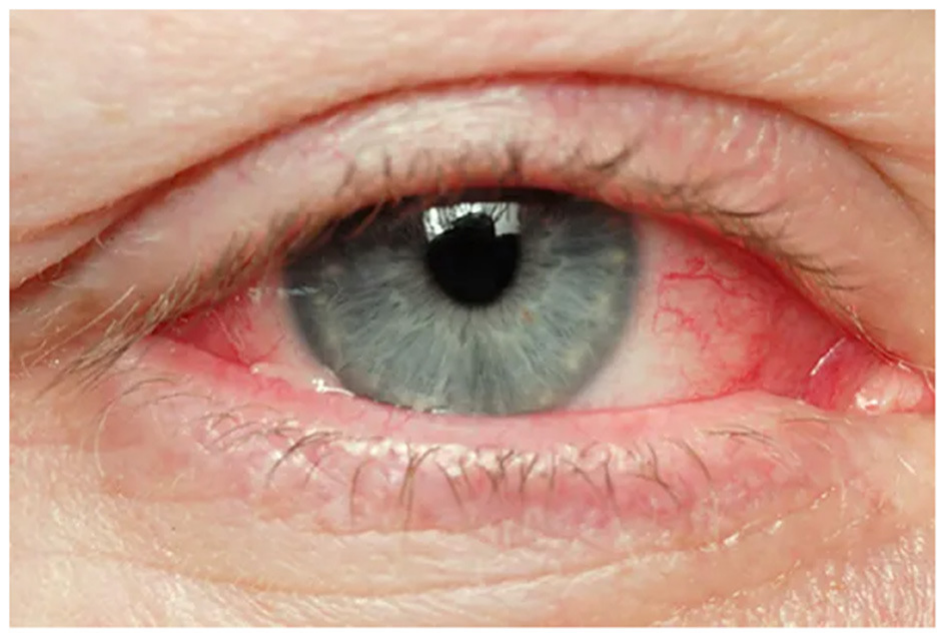
Eye redness. Reproduced from WebMD, LLC, https://www.webmd.com/eye-health/eye-health-conjunctivitis#1-4.

**Figure 4:**
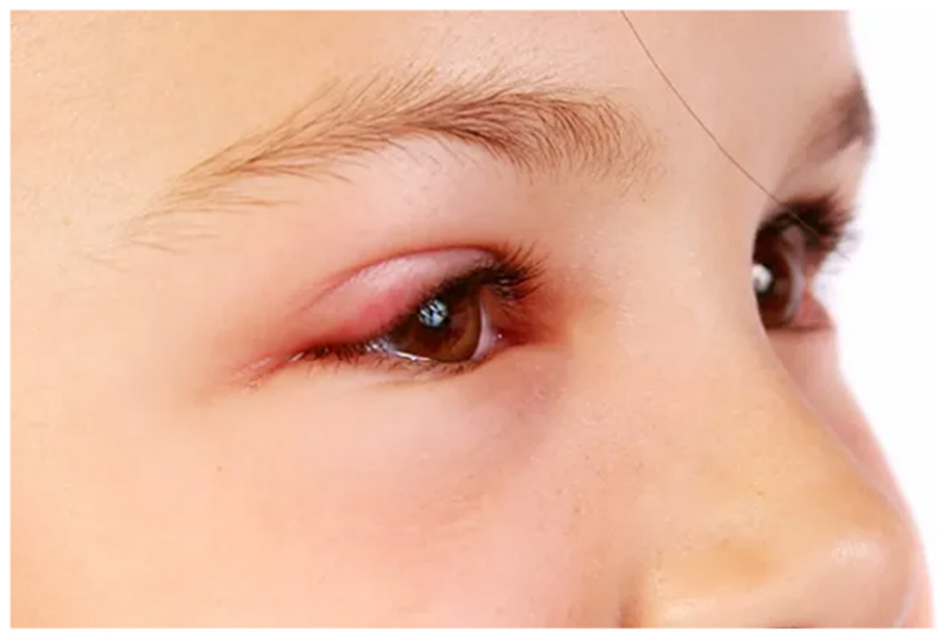
Swelling in the eye. Reproduced from WebMD, LLC, https://www.webmd.com/eye-health/eye-health-conjunctivitis#1-4.

**Figure 5:**
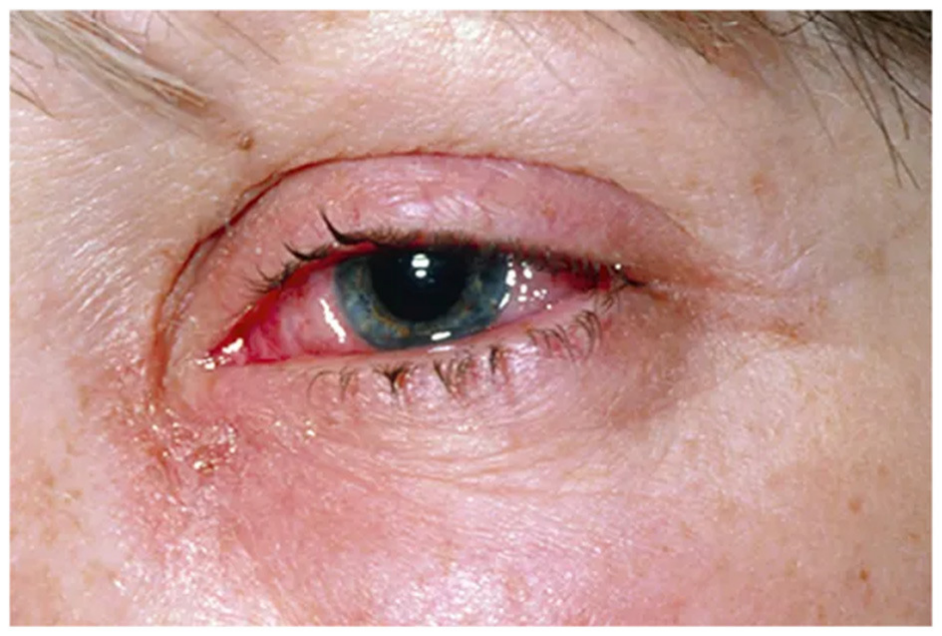
Itchy or burning eyes. Reproduced from WebMD, LLC, https://www.webmd.com/eye-health/eye-health-conjunctivitis#1-4.

**Figure 6:**
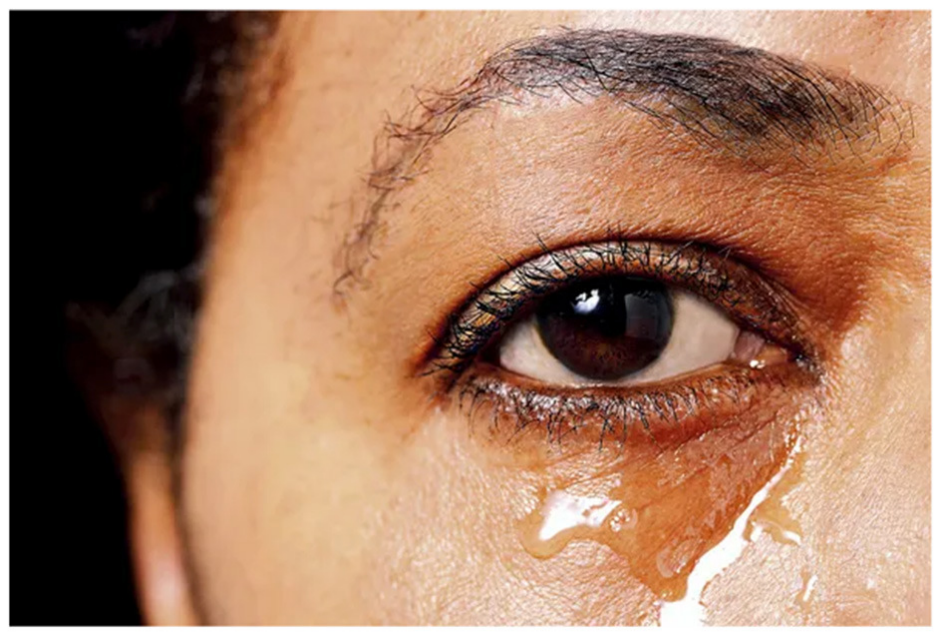
Lots of tearing. Reproduced from WebMD, LLC, https://www.webmd.com/eye-health/eye-health-conjunctivitis#1-4.

Visual depiction of symptoms is given in Figures 1–6 (https://www.webmd.com/eye-health/eye-health-conjunctivitis#1-4).

The appropriate course of medication for conjunctivitis is contingent upon the specific etiology.

- Buzzing conjunctivitis: Generally exhibits a self-limiting course, with spontaneous resolution occurring throughout a period of around one to two weeks. Over-the-counter lubricating eye drops can help relieve discomfort, and cold compresses may reduce swelling.
- Bacterial conjunctivitis: The administration of antiseptic eye drops or lotion is necessary and should be administered by a qualified wellness services professional. It is essential to complete the full course of antibiotics even if symptoms improve.
- Allergic conjunctivitis: Antihistamine eye drops, oral antihistamines, or allergy medications may help relieve symptoms. Avoiding allergens is also crucial.
- Irritant conjunctivitis: Removing the irritant and using artificial tears can alleviate symptoms.

It is mandatory to practice good hygiene to mitigate the transmission of contagious pink eye:

- Washing of hands frequently, especially before touching eyes or face.
- Avoid sharing towels, pillows, or eye makeup with someone who has pink eye.
- If anyone has pink eye, avoid close contact with the infected person until he/she no longer contagious.

This article describes an outstanding multi-attribute group decision-making (MAGDM) method, the 2TL*T*-SF-WASPAS method, which uses combined optimality values to arrive at the outcome. Additionally, the suggested method includes 2-tuple linguistic (2TL) evaluations that assist the experts in making judgments using language phrases. Through a case study for choosing the appropriate medication for pink eye infection, we argue for the suggested method.

The article aims to provide a comprehensive overview of the underlying motivations that drive the research conducted in this study.

1. The *T*-spherical fuzzy set (*T*-SFS) (Mahmood et al., 2019) is a more generalized potent of the current fuzzy set (FS) theories and is able to accommodate and manage far bigger amounts of data than the FS and its related expansions. Three degrees make up the *T*-SFS (referred to as membership degree (MD), abstinence degree (AD), and non-membership degree (NMD)), which enables the decision-makers (DMs) to communicate more effectively about their points of view. These important characteristics of the *T*-SFS encourages us to use it in our research study.
2. Experts are finding that MAGDM issues are getting harder over time. The DMs find it difficult at times to express their opinions and preferences in numbers. To qualitatively express their evaluation information, linguistic terms (LTs) give DMs a wide range of options. Additionally, the 2TL term set (Herrera and Martínez, 2000) improves upon the shortcomings of the traditional LT set. The values in the 2TL model are represented by a pair of components as (ℑ, ♮), where ℑ denotes the LT and ♮ known as its symbolic translation.
3. We use the concept of a 2TL*T*-SFS (Akram et al., 2023c) because of the aforementioned characteristics of 2TL representation model and *T*-SFS. The *T*-SFS and the 2TL terms have been generalized to become the 2TL*T*-SFS. The traits of *T*-SFS and 2TL terms are combined in the 2TL*T*-SFS. To appropriately describe data, 2TL*T*-SFS expands the space and represents MDs, ADs, and NMDs as 2-tuples.
4. Both the Schweizer-Sklar weighted average (SSWA) and the Schweizer-Sklar weighted geometric (SSWG) operators are adaptable and practical instruments for group decision-making. In MAGDM situations, these operators can combine data quite effectively. These assertions encouraged us to develop SSWA and SSWG operators for the 2TL*T*-SFS.
5. WASPAS method (Zavadskas et al., 2012) is a well-established decision-making approach which has been used by many researchers in the literature for different decision-making scenarios (Arslan and Cebi, 2024; Verma and Álvarez-Miranda, 2024; Goyal and Rani, 2024). To address group decision-making challenges in selecting the optimal medication for pink eye infection under the 2TL*T*-SFS framework, an enhanced approach employing the WASPAS method offers a scientifically robust and effective solution. This strategy leverages the integration of multiple decision-making criteria, enhancing accuracy and reliability in the evaluation of treatment options.

Now, we will discuss the contributions of the paper.

1. The 2TL*T*-SFSSWA and the 2TL*T*-SFSSWG operators are formulated and their significant and prominent properties are described.
2. We construct the WASPAS approach for 2TL*T*-SFS to deal with the difficulties in MAGDM situations. Based on past research, we determine that the WASPAS method must be built within the 2TL*T*-SFS context. As a result, we decide to develop a WASPAS approach for the 2TL*T*-SFS.
3. We create a framework to represent the steps of the suggested plan. The accompanying framework makes the process clear and explains each and every stage of the suggested strategy.
4. We use a numerical case to illustrate how the formulated technique can be used. The use of the suggested methodology in the field of medicine is something we research. In the case study that is offered, the optimum medication for treating pink eye infection is chosen.
5. To demonstrate the success of the suggested methodology, we outline a parameter analysis. By conducting a comparison, we prove the authenticity and superiority of the conventional approach.

Section 2 contains an overview of the study's literature. Section 3 goes over the fundamentals of the 2TL terms, 2TL*T*-SFS, SSWA operator, SSWG operator. In Section 4, we explore the 2TL*T*-SFSSWA and 2TL*T*-SFSSWG operators and describe their important and prominent properties along with their operational laws. Section 5 discusses an enhanced WASPAS approach based on the 2TL*T*-SFS as well as a completely designed flowchart to better show the formulated technique. Section 6 applies the suggested method to a case study for pink eye infection, complete with parameter and comparison analyses. Finally, in Section 7, we discuss our conclusions, study implications, limitations, and future research.

## 2 Literature review

MAGDM is a sophisticated decision-making science that focuses on how to choose the best choice through opinion expression, knowledge synthesis, and the analytical ranking of fuzzy data. Because humans are inherently uncertain when confronted with complicated issues, it is difficult for DMs to deliver precise evaluation results. MAGDM is an exciting area of research that tries to develop methods and rationales for selecting the best candidate from a pool of probable candidates. Numerous academics have looked into its approaches, and the results of their research have been effectively utilized to address a wide range of challenges associated with making choices. To overcome this obstacle, Zadeh (Zadeh, 1965) first developed FS, it has subsequently proven an important instrument in the course of making choices, especially in situations containing vague and confusing content. Many real-world situations are fraught with vague and ambiguity, making it imprecise for DMs to form judgments. Uncertainties represent important challenges in dealing with incomplete or ambiguous data, from environmental impact assessment to clinical diagnostics, and all in between. Ambiguity is a common occurrence in everyday life, causing a variety of problems in many circumstances. As a result, it is critical to address doubts caused by ambiguity because there are numerous approaches to resolving vagueness or ambiguity. To deal with ambiguity in real-world problems, academics have created new FS models such as intuitionistic FS (Atanassov, 1986), Pythagorean FS (Yager, 2013), *q*-rung orthopair FS (*q*-ROFS) (Yager, 2016). In order to solve MAGDM, Abbas et al. (2024c) suggested a novel approach to decision-making that combines QUALIFLEX and VIKOR. Using sensitivity and comparative analysis, the VIKOR-QUALIFLEX MAGDM method was used to choose the best bike-sharing recycling supplier, proving its availability and applicability.

The *T*-SFS (Mahmood et al., 2019) is the most sophisticated generalization of the *q*-ROFS, which can handle ambiguity, fuzziness, and uncertainty in relation to four dimensions, namely positivism (affirmation), pessimism (negation), neutrality (abstention), and refusal (rejection). To assess the veracity of confusing data, 2TL terminology is used. Herrera and Martínez (2000) suggested the 2TL variable as an innovative and efficient method of representing information in the presence of ambiguity and uncertainty. It is regarded as a suitable technique to convey uncertain information provided by DMs since it uses a 2-tuple derived from linguistic words to represent the linguistic assessment information in an uncertain environment. Therefore, a 2TL variable could be a useful tool to convey the experts' opinions when there is ambiguity and uncertainty in the dependence evaluation. (Abbas et al., 2024a) used partitioning techniques to divide criteria into discrete groups in order to study MAGDM. They developed an integrated weighting approach and generated partitioned Hamy mean and partitioned dual Hamy mean operators in the *q*-ROF2L environment. To identify the most optimal choice for manufacturing a linear delta robot, Akram et al. (2023a) constructed an approach using the 2TL*T*-SF numbers. Akram et al. (2023b) offered a broad framework for expressing and computing qualitative evaluation using the combination of *T*-SFS and 2TL representation model. Akram et al. (2023c) developed the 2TL*T*-SFS, which generalize *T*-SFS using 2TL terms, to address uncertainty surrounding MAGDM scenarios. In the context of the cognitive data supplied in the hospital evaluation process, Naz et al. (2023a) suggested a MAGDM method employing a 2TL*T*-SFS. In the context of *t*-norm and *t*-conorm, Mahmood et al. (2023) developed the innovative idea of the *T*-spherical 2TL set and its operational laws. For the purpose of identifying ideal data mining tactics, considered an essential component of contemporary study on making decisions, Naz et al. (2023b) presented a MAGDM method that was implemented in a *T*-SF setting. The application of SFS to expert judgment and uncertainty management was the main focus of that study presented by Ali (Ali, 2024a). Aczel-Alsina operational laws were used to formulate averaging and geometric operators, incorporating prioritization degrees based on SF information. Ali (2024b) investigated the expression of assessment information grades using linguistic SFS. He created extended power operators and presented novel operating rules based on Archimedean copula and co-copula.

The utilization of aggregation operators (AOs) is gaining significance in the field of multi-attribute decision-making (MADM), management for mechanisms, support, and cognitive reasoning. The fundamental attribute that distinguishes AOs is their ability to integrate numerous components in order to generate a singular response that encapsulates the comprehensive result of the choice-making procedure. Schweizer-Sklar (SS) ventures are extremely adaptable and preferable to other ventures due to their variable parameter (Deschrijver and Kerre, 2002). The recommended AOs are better at solving highly complex MAGDM challenges because they can exclude undesirable details that are excessively substantial or shallow. Gayen et al. (2023) originally established various operations, namely addition, multiplication, scalar multiplication, exponential laws based on the SS class of *t*-conorms, and *t*-norms, in order to make the dual hesitant *q*-ROF aggregation information process adaptable. With the help of the cubic *m*-polar FS and the *t*-norm and *t*-conorm, Kausar et al. (2023) created the SS AO, which is based on the cubic *m*-polar FS. Liu D. et al. (2023) expanded the SS *t*-norm and *t*-conorm sets to *q*-rung orthopair normal fuzzy number and formulated the SS operational rules. Pamucar et al. (2023) introduced a unique structure for evaluating metaverse incorporation options using a variety of criteria founded on ordinal prioritization. The structure incorporates FS theory and SS norms. Based on the *q*-rung orthopair probabilistic hesitant fuzzy SS power-weighted Hamy mean operator, Chen et al. (2023) suggested a MADM approach. In the context of a bipolar complex fuzzy (BCF) set, Mahmood and ur Rehman (2023) researched operational laws based on the SS *t*-norm and *t*-conorm and created AOs based on these derived operational laws. On the basis of picture fuzzy information, Hussain et al. (2022) created a class of novel methods, including the picture fuzzy SS prioritized average and the picture fuzzy SS prioritized geometric operators. In order to deal with situations where criteria were separated into separate and related parts, Azeem et al. (2024) suggested two new operators: complex Fermatean fuzzy weighted partitioned Maclaurin symmetric mean and complex Fermatean fuzzy partitioned Maclaurin symmetric mean. Abbas et al. (2024b) suggested a novel method that used the *q*-ROF2TLS to help medical professionals prioritize patients. To evaluate priority degrees and relationships, it combines Maclaurin symmetric mean operators with prioritized averaging.

Due to the variety of MADM problems, numerous strategies have needed to be developed in order to address them in a variety of unpredictable contexts. One of the most recent MADM techniques presented by Zavadskas et al. (2012) is the WASPAS, which is founded on utility theory. This framework takes parts from both the weighted product model (Fishburn, 1967) and the weighted sum model (Bridgman, 1922). It takes the conclusions of the weighted sum model and the weighted product model to make a collective standardized categories measure. This amount is then utilized in ranking the options. Rendering decisions using the WASPAS method is very quick and good. The ease of processing, the predictability of the results, and the high sensitivity it possesses to the phenomenon of alternative order inversion are among its primary advantages. Despite all of its benefits, the WASPAS approach cannot manage information that is imprecise, hazy, or ambiguous. Additionally, it disregards the facts that DMs neutralize or reject. To clarify the MADM issues with BCF data, Liu P. et al. (2023) created an integrated BCF-CRITIC-WASPAS technique. By combining the Fermatean fuzzy-WASPAS and the triangular fuzzy logarithm methodology of additive weights, Sicakyüz (2023) proposed a newly created hybrid MADM approach. Alrasheedi et al. (2023) created an integrated WASPAS method based on the SWARA criterion weighting model to solve MAGDM issues in intuitionistic fuzzy environments. Rao et al. (2023) developed a consensus-based WASPAS strategy to handle the problem of MADM difficulties with Fermatean fuzzy data. Barbara et al. (2023) discussed the results of the WASPAS WEB project, which aims to provide DMs with a simple means for using the WASPAS approach. In order to effectively address decision-making issues on interval-valued Pythagorean FS, Al-Barakati et al. (2022) proposed an integrated method based on the WASPAS method.

## 3 Preliminaries

This section provides a concise summary of the key concepts pertaining to the 2TL terms, the *T*-SFS, and the 2TL*T*-SFS, which will facilitate the understanding of subsequent sections.

Definition 1. (Herrera and Martínez, 2000) Let *S* = {ℑ_ı_|ı = 0, 1, …, ‡} be characterized as a completely structured and finite linguistic term set (LTS) with cardinality ‡ + 1 and let ℷ ∈ [0, ‡] be the quantitative quantity. Let ı ∈ {0, 1, …, ‡} and ♮ ∈ [−0.5, 0.5) be two outcomes, then ♮ is referred to by a symbolic translation, being a quantitative number that represents the quantity of translation from the first outcome ℷ to the closest indexing labeling ı. In this case, ı is indicated as round(ℷ), while ♮ = ℷ − ı.

The 2TL concept is elucidated by employing the translation tool described earlier to bridge between quantitative data and 2-tuples.

Definition 2. (Herrera and Martínez, 2000) Consider a discrete LTS defined as *S* = {ℑ_ı_, |, ı = 0, 1, …, ‡} and let ℷ be a numeric value in the range [0, ‡] that represents the outcome of a symbolic aggregating process. Below, a 2-tuple that conveys the same information as ℷ:


$$\begin{array}{l} \Delta :\left[0,‡\right]\to \bar{S},\\ \Delta \left(ℷ\right)=\left({ℑ}_{\text{round}\left(ℷ\right)},ℷ-ı;\right), \end{array}$$

the expression $\bar{S}$ = S * (-0.5, 0.5), round(.) refers to defining $\bar{S}$ as the product of set *S* and the interval (−0.5, 0.5), and round(.) indicates the standard rounding function that maps a real value ℷ to the nearest integer ı within the set {0, 1, …, ‡}. Finally, the term ♮ = ℷ − ı signifies the concept of symbolic translation.

Remark 1. (Herrera and Martínez, 2000) As previously mentioned, the mapping denoted by Δ is a one-to-one function, which means it has an inverse. Consequently, there exists an inverse function, denoted as Δ^−1^, that operates on a set denoted by $\bar{S}$ and maps it to the interval [0, ‡]. This inverse function yields the same precise outcome for any given pair of values denoted as ℑ_ı_ and ♮, the result ℷ, which falls within the range [0, ‡] and is also a real number, corresponds to an identical 2-tuple. In other words, $\Delta^{-1}\left({ℑ}_{ı;},♮\right)=ℷ$ is equivalent to the mathematical expression ℷ = ı+♮.

Remark 2. The 2TL term is derived from a LT ℑ_ı_ through a straightforward process of augmenting it with a symbolic translation of zero. This can be expressed as follows: If ℑ_ı_ belongs to the set *S*, then the pair (ℑ_ı_, 0) also belongs to the set *S* × [−0.5, 0.5).

Remark 3. (Herrera and Martínez, 2000) conducted a comparison of 2TL information using the standard lexicographic ordering, as described in their study.

Definition 3. Let (ℑ_ı_, ♮_ı_) and (ℑ_φ_, ♮_φ_) be two 2TL terms.

– if ı < φ⇒(ℑ_ı_, ♮_ı_) < (ℑ_φ_, ♮_φ_).
– if ı = φ then,
  (1) if ♮_ı_ = ♮_φ_ ⇒ (ℑ_ı_, ♮_ı_) = (ℑ_φ_, ♮_φ_);
  (2) if ♮_ı_ < ♮_φ_ ⇒ (ℑ_ı_, ♮_ı_) < (ℑ_φ_, ♮_φ_);
  (3) if ♮_ı_ > ♮_φ_ ⇒ (ℑ_ı_, ♮_1_) > (ℑ_φ_, ♮_2_).

Definition 4. (Herrera and Martínez, 2000) Consider the finite cardinality set *S*, defined as *S* = {ℑ_ı_|ı = 0, 1, …, ‡}, where ‡ + 1 represents the number of elements in the set. In this context, the negative operator for a 2-tuple is as follows:


$$\begin{array}{l} Neg\left({ℑ}_{ı;},♮\right)=\Delta \left(‡-\left(\Delta^{-1}\left({ℑ}_{ı;},♮\right)\right)\right). \end{array}$$

Definition 5. (Mahmood et al., 2019) In the context of any universal set denoted as L, a *T*-SFS within L takes the form of


T={(†,(𝔯(†),𝔱(†),𝔩(†)))∣†∈L},

in the context, 𝔯(†), 𝔱(†), 𝔩(†) ∈ [0, 1] represent the MD, AD, and NMD (respectively) of a specific element † within the set L, it is necessary to satisfy the condition that 0 ≤ 𝔯^*q*^(†)+𝔱^*q*^(†)+𝔩^*q*^(†) ≤ 1 for all values of *q* greater than or equal to 1. We can refer to the value $r\left(†\right)=\sqrt[{q}]{1-\left({𝔯}^{q}\left(†\right)+{𝔱}^{q}\left(†\right)+{𝔩}^{q}\left(†\right)\right)}$ as the degree of refusal of † in the context of set L.

To simplify matters, this triplet (𝔯(†), 𝔱(†), 𝔩(†)) can also be termed as a *T*-SFN.

Definition 6. (Akram et al., 2023c) Consider a universal set denoted as L. Within this set, a 2TL*T*-SFS Υ is defined as follows:


(1) $$\begin{array}{l} \Upsilon =\left\{\langle †,\left(\left({ℑ}_{𝔯}\left(†\right),R\left(†\right)\right),\left({ℑ}_{𝔱}\left(†\right),T\left(†\right)\right),\left({ℑ}_{𝔩}\left(†\right),L\left(†\right)\right)\right)\rangle |†\in \text{L}\right\}, \end{array}$$

where $\left({ℑ}_{𝔯}\left(†\right),R\left(†\right)\right),\left({ℑ}_{𝔱}\left(†\right),T\left(†\right)\right)$ and $\left({ℑ}_{𝔩}\left(†\right),L\left(†\right)\right)$ represent the positive, neutral, and negative membership degrees, respectively. It is required that -0.5≤R(†),T(†),L(†)<0.5, $0\leq \Delta^{-1}\left({ℑ}_{𝔯}\left(†\right),R\left(†\right)\right)\leq ‡$, $0\leq \Delta^{-1}\left({ℑ}_{𝔱}\left(†\right),T\left(†\right)\right)\leq ‡$, $0\leq \Delta^{-1}\left({ℑ}_{𝔩}\left(†\right),L\left(†\right)\right)\leq ‡$ where *S* = {ℑ_ı_|ı = 0, 1, …, ‡} is a LTS with odd cardinality and


$$\begin{array}{l} 0\leq {\left(\Delta^{-1}\left({ℑ}_{𝔯}\left(†\right),R\left(†\right)\right)\right)}^{q}+{\left(\Delta^{-1}\left({ℑ}_{𝔱}\left(†\right),T\left(†\right)\right)\right)}^{q}\\ +{\left(\Delta^{-1}\left({ℑ}_{𝔩}\left(†\right),L\left(†\right)\right)\right)}^{q}\leq {‡}^{q}. \end{array}$$

Regarding ease of use, $\Upsilon =\left(\left({ℑ}_{𝔯},R\right),\left({ℑ}_{𝔱},T\right),\left({ℑ}_{𝔩},L\right)\right)$ is called the 2TL*T*-SFN, where


$$0\leq \Delta^{-1}\left({ℑ}_{𝔯},R\right),\Delta^{-1}\left({ℑ}_{𝔱},T\right),\Delta^{-1}\left({ℑ}_{𝔩},L\right)\leq ‡,$$

and


$$0\leq {\left(\Delta^{-1}\left({ℑ}_{𝔯},R\right)\right)}^{q}+{\left(\Delta^{-1}\left({ℑ}_{𝔱},T\right)\right)}^{q}+{\left(\Delta^{-1}\left({ℑ}_{𝔩},L\right)\right)}^{q}\leq {‡}^{q}.$$

To conduct a comparison between two 2TL*T*-SFNs, the score and accuracy functions can be determined according to the accompanying description:

Definition 7. (Akram et al., 2023c) Let $\Upsilon =\left(\left({ℑ}_{𝔯},R\right),\left({ℑ}_{𝔱},T\right),\left({ℑ}_{𝔩},L\right)\right)$ be a 2TL*T*-SFN. Then the score function *Sc* can be defined as:


(2) $$\begin{array}{l} Sc\left(\Upsilon \right)=\Delta \left(\frac{‡}{2}\left(1+{\left(\frac{\Delta^{-1}\left({ℑ}_{𝔯},R\right)}{‡}\right)}^{q}-{\left(\frac{\Delta^{-1}\left({ℑ}_{𝔩},L\right)}{‡}\right)}^{q}\right)\right), \end{array}$$

and the accuracy function *Ac* can be defined as:


(3) $$\begin{array}{l} Ac\left(\Upsilon \right)=\Delta \left(‡\left({\left(\frac{\Delta^{-1}\left({ℑ}_{𝔯},R\right)}{‡}\right)}^{q}+{\left(\frac{\Delta^{-1}\left({ℑ}_{𝔩},L\right)}{‡}\right)}^{q}\right)\right). \end{array}$$

Definition 8. (Akram et al., 2023c) Let $\Upsilon_{1}=\left(\left({ℑ}_{{𝔯}_{1}},R_{1}\right),\left({ℑ}_{{𝔱}_{1}},T_{1}\right),\left({ℑ}_{{𝔩}_{1}},L_{1}\right)\right)$, and $\Upsilon_{2}=\left(\left({ℑ}_{{𝔯}_{2}},R_{2}\right),\left({ℑ}_{{𝔱}_{2}},T_{2}\right),\left({ℑ}_{{𝔩}_{2}},L_{2}\right)\right)$ be two 2TL*T*-SFNs. The evaluation of the two 2TL*T*-SFNs can be conducted by utilizing their respective score and accuracy functions in the following manner:

- If *Sc*(Υ_1_) < *Sc*(Υ_2_), then Υ_1_ ≺ Υ_2_;
- If *Sc*(Υ_1_) > *Sc*(Υ_2_), then Υ_1_ ≻ Υ_2_;
- If *Sc*(Υ_1_) = *Sc*(Υ_2_), then
  (a) If *Ac*(Υ_1_) < *Ac*(Υ_2_), then Υ_1_ ≺ Υ_2_;
  (b) If *Ac*(Υ_1_) > *Ac*(Υ_2_), then Υ_1_ ≻ Υ_2_;
  (c) If *Ac*(Υ_1_) = *Ac*(Υ_2_), then Υ_1_ ~ Υ_2_.

Definition 9. (Akram et al., 2023c) Let $\Upsilon =\left(\left({ℑ}_{𝔯},R\right),\left({ℑ}_{𝔱},T\right),\left({ℑ}_{𝔩},L\right)\right)$, $\Upsilon_{1}=\left(\left({ℑ}_{{𝔯}_{1}},R_{1}\right),\left({ℑ}_{{𝔱}_{1}},T_{1}\right),\left({ℑ}_{{𝔩}_{1}},L_{1}\right)\right)$, and $\Upsilon_{2}=\left(\left({ℑ}_{{𝔯}_{2}},R_{2}\right),\left({ℑ}_{{𝔱}_{2}},T_{2}\right),\left({ℑ}_{{𝔩}_{2}},L_{2}\right)\right)$ be three 2TL*T*-SFNs, *q* ≥ 1 and σ > 0. Then

1.
$$\begin{array}{l} \Upsilon_{1}⊕\Upsilon_{2}\\ =\left(\begin{array}{c} \Delta \left(‡\sqrt[{q}]{1-\left(1-{\left(\frac{\Delta^{-1}\left({ℑ}_{{𝔯}_{1}},R_{1}\right)}{‡}\right)}^{q}\right)\left(1-{\left(\frac{\Delta^{-1}\left({ℑ}_{{𝔯}_{2}},R_{2}\right)}{‡}\right)}^{q}\right)}\right),\\ \Delta \left(‡\left(\frac{\Delta^{-1}\left({ℑ}_{{𝔱}_{1}},T_{1}\right)}{‡}\right)\left(\frac{\Delta^{-1}\left({ℑ}_{{𝔱}_{2}},T_{2}\right)}{‡}\right)\right),\\ \Delta \left(‡\left(\frac{\Delta^{-1}\left({ℑ}_{{𝔩}_{1}},L_{1}\right)}{‡}\right)\left(\frac{\Delta^{-1}\left({ℑ}_{{𝔩}_{2}},L_{2}\right)}{‡}\right)\right) \end{array}\right); \end{array}$$
2.
$$\begin{array}{l} \Upsilon_{1}⊗\Upsilon_{2}=\\ \left(\begin{array}{c} \Delta \left(‡\left(\frac{\Delta^{-1}\left({ℑ}_{{𝔯}_{1}},R_{1}\right)}{‡}\right)\left(\frac{\Delta^{-1}\left({ℑ}_{{𝔯}_{2}},R_{2}\right)}{‡}\right)\right),\\ \Delta \left(‡\sqrt[{q}]{1-\left(1-{\left(\frac{\Delta^{-1}\left({ℑ}_{{𝔱}_{1}},T_{1}\right)}{‡}\right)}^{q}\right)\left(1-{\left(\frac{\Delta^{-1}\left({ℑ}_{{𝔱}_{2}},T_{2}\right)}{‡}\right)}^{q}\right)}\right),\\ \Delta \left(‡\sqrt[{q}]{1-\left(1-{\left(\frac{\Delta^{-1}\left({ℑ}_{{𝔩}_{1}},L_{1}\right)}{‡}\right)}^{q}\right)\left(1-{\left(\frac{\Delta^{-1}\left({ℑ}_{{𝔩}_{2}},L_{2}\right)}{‡}\right)}^{q}\right)}\right) \end{array}\right); \end{array}$$
3.
$$\begin{array}{l} \sigma \Upsilon =\left(\begin{array}{c} \Delta \left(‡\sqrt[{q}]{1-{\left(1-{\left(\frac{\Delta^{-1}\left({ℑ}_{𝔯},R\right)}{‡}\right)}^{q}\right)}^{\sigma }}\right),\\ \Delta \left(‡{\left(\frac{\Delta^{-1}\left({ℑ}_{𝔱},T\right)}{‡}\right)}^{\sigma }\right),\\ \Delta \left(‡{\left(\frac{\Delta^{-1}\left({ℑ}_{𝔩},L\right)}{‡}\right)}^{\sigma }\right) \end{array}\right); \end{array}$$
4.
$$\begin{array}{l} \Upsilon^{\sigma }=\left(\begin{array}{c} \Delta \left(‡{\left(\frac{\Delta^{-1}\left({ℑ}_{𝔯},R\right)}{‡}\right)}^{\sigma }\right),\\ \Delta \left(‡\sqrt[{q}]{1-{\left(1-{\left(\frac{\Delta^{-1}\left({ℑ}_{𝔱},T\right)}{‡}\right)}^{q}\right)}^{\sigma }}\right),\\ \Delta \left(‡\sqrt[{q}]{1-{\left(1-{\left(\frac{\Delta^{-1}\left({ℑ}_{𝔩},L\right)}{‡}\right)}^{q}\right)}^{\sigma }}\right) \end{array}\right). \end{array}$$

## 4 The 2TL*T*-SF Schweizer-Sklar AOs

In this part, we present two forms of weighting informational AOs, namely 2TL*T*-SFSSWA and 2TL*T*-SFSSWG operators along with the 2TL*T*-SF Schweizer-Sklar operational laws. The idempotency, monotonicity, and boundedness features of the two suggested AOs are also discussed.

Definition 10. Let $\Upsilon =\left(\left({ℑ}_{𝔯},R\right),\left({ℑ}_{𝔱},T\right),\left({ℑ}_{𝔩},L\right)\right)$, $\Upsilon_{1}=\left(\left({ℑ}_{{𝔯}_{1}},R_{1}\right),\left({ℑ}_{{𝔱}_{1}},T_{1}\right),\left({ℑ}_{{𝔩}_{1}},L_{1}\right)\right)$, and $\Upsilon_{2}=\left(\left({ℑ}_{{𝔯}_{2}},R_{2}\right),\left({ℑ}_{{𝔱}_{2}},T_{2}\right),\left({ℑ}_{{𝔩}_{2}},L_{2}\right)\right)$ be three 2TL*T*-SFNs, *q* ≥ 1 and σ > 0. Then, the 2TL*T*-SF Schweizer-Sklar operational laws are defined as:

1.
$$\begin{array}{l} \Upsilon_{1}{⊕}_{s}\Upsilon_{2}\\ =\left(\begin{array}{c} \Delta \left(‡\sqrt[{q}]{1-{\left({\left(1-{\left(\frac{\Delta^{-1}\left({ℑ}_{{𝔯}_{1}},R_{1}\right)}{‡}\right)}^{q}\right)}^{k}+{\left(1-{\left(\frac{\Delta^{-1}\left({ℑ}_{{𝔯}_{2}},R_{2}\right)}{‡}\right)}^{q}\right)}^{k}-1\right)}^{\frac{1}{k}}}\right),\\ \Delta \left(‡{\left({\left(\frac{\Delta^{-1}\left({ℑ}_{{𝔱}_{1}},T_{1}\right)}{‡}\right)}^{qk}+{\left(\frac{\Delta^{-1}\left({ℑ}_{{𝔱}_{2}},T_{2}\right)}{‡}\right)}^{qk}-1\right)}^{\frac{1}{qk}}\right),\\ \Delta \left(‡{\left({\left(\frac{\Delta^{-1}\left({ℑ}_{{𝔩}_{1}},L_{1}\right)}{‡}\right)}^{qk}+{\left(\frac{\Delta^{-1}\left({ℑ}_{{𝔩}_{2}},L_{2}\right)}{‡}\right)}^{qk}-1\right)}^{\frac{1}{qk}}\right) \end{array}\right); \end{array}$$
2.
$$\begin{array}{l} \Upsilon_{1}{⊗}_{s}\Upsilon_{2}\\ =\left(\begin{array}{c} \Delta \left(‡{\left({\left(\frac{\Delta^{-1}\left({ℑ}_{{𝔯}_{1}},R_{1}\right)}{‡}\right)}^{qk}+{\left(\frac{\Delta^{-1}\left({ℑ}_{{𝔯}_{2}},R_{2}\right)}{‡}\right)}^{qk}-1\right)}^{\frac{1}{qk}}\right),\\ \Delta \left(‡\sqrt[{q}]{1-{\left({\left(1-{\left(\frac{\Delta^{-1}\left({ℑ}_{{𝔱}_{1}},T_{1}\right)}{‡}\right)}^{q}\right)}^{k}+{\left(1-{\left(\frac{\Delta^{-1}\left({ℑ}_{{𝔱}_{2}},T_{2}\right)}{‡}\right)}^{q}\right)}^{k}-1\right)}^{\frac{1}{k}}}\right),\\ \Delta \left(‡\sqrt[{q}]{1-{\left({\left(1-{\left(\frac{\Delta^{-1}\left({ℑ}_{{𝔩}_{1}},L_{1}\right)}{‡}\right)}^{q}\right)}^{k}+{\left(1-{\left(\frac{\Delta^{-1}\left({ℑ}_{{𝔩}_{2}},L_{2}\right)}{‡}\right)}^{q}\right)}^{k}-1\right)}^{\frac{1}{k}}}\right) \end{array}\right); \end{array}$$
3.
$$\begin{array}{l} \sigma \Upsilon \\ =\left(\begin{array}{c} \Delta \left(‡\sqrt[{q}]{1-{\left(\sigma {\left(1-{\left(\frac{\Delta^{-1}\left({ℑ}_{𝔯},R\right)}{‡}\right)}^{q}\right)}^{k}-\left(\sigma -1\right)\right)}^{\frac{1}{qk}}}\right),\\ \Delta \left(‡{\left(\left(\sigma {\left(\frac{\Delta^{-1}\left({ℑ}_{𝔱},T\right)}{‡}\right)}^{qk}\right)-\left(\sigma -1\right)\right)}^{\frac{1}{qk}}\right),\\ \Delta \left(‡{\left(\left(\sigma {\left(\frac{\Delta^{-1}\left({ℑ}_{𝔩},L\right)}{‡}\right)}^{qk}\right)-\left(\sigma -1\right)\right)}^{\frac{1}{qk}}\right) \end{array}\right); \end{array}$$
4.
$$\begin{array}{l} \Upsilon^{\sigma }\\ =\left(\begin{array}{c} \Delta \left(‡{\left(\left(\sigma {\left(\frac{\Delta^{-1}\left({ℑ}_{𝔯},R\right)}{‡}\right)}^{qk}\right)-\left(\sigma -1\right)\right)}^{\frac{1}{qk}}\right),\\ \Delta \left(‡\sqrt[{q}]{1-{\left(\sigma {\left(1-{\left(\frac{\Delta^{-1}\left({ℑ}_{𝔱},T\right)}{‡}\right)}^{q}\right)}^{k}-\left(\sigma -1\right)\right)}^{\frac{1}{qk}}}\right),\\ \Delta \left(‡\sqrt[{q}]{1-{\left(\sigma {\left(1-{\left(\frac{\Delta^{-1}\left({ℑ}_{𝔩},L\right)}{‡}\right)}^{q}\right)}^{k}-\left(\sigma -1\right)\right)}^{\frac{1}{qk}}}\right) \end{array}\right); \end{array}$$

Definition 11. Let $\Upsilon_{\varphi }=\left(\left({ℑ}_{{𝔯}_{\varphi }},R_{\varphi }\right),\left({ℑ}_{{𝔱}_{\varphi }},T_{\varphi }\right),\left({ℑ}_{{𝔩}_{\varphi }},L_{\varphi }\right)\right)\left(\varphi =1,2,\ldots ,ñ\right)$ be an accumulation of 2TL*T*-SFNs. The 2TL*T*-SFSSWA operator represents a mapping from *T*^ñ^ to *T*, characterized by the following


(4) $$\begin{array}{l} \text{2TL}T\text{-SFSSWA}\left(\Upsilon_{1},\Upsilon_{2},\ldots ,\Upsilon_{ñ}\right)={⊕}_{\varphi =1}^{ñ}{♭}_{\varphi }\Upsilon_{\varphi }, \end{array}$$

in which *T* is the set of 2TL*T*-SFNs, $♭={\left({♭}_{1},{♭}_{2},\ldots ,{♭}_{ñ}\right)}^{T}$ is the weight vector of Υ_φ_(φ = 1, 2, …, ñ), such that ♭_φ_ ∈ [0, 1] and $\overset{ñ}{\underset{\varphi =1}{\sum }}{♭}_{\varphi }=1.$

Theorem 1. Let $\Upsilon_{\varphi }=\left(\left({ℑ}_{{𝔯}_{\varphi }},R_{\varphi }\right),\left({ℑ}_{{𝔱}_{\varphi }},T_{\varphi }\right),\left({ℑ}_{{𝔩}_{\varphi }},L_{\varphi }\right)\right)\left(\varphi =1,2,\ldots ,ñ\right)$ be an accumulation of 2TL*T*-SFNs with weight vector $♭={\left({♭}_{1},{♭}_{2},\ldots ,{♭}_{ñ}\right)}^{T}$, such that ♭_φ_ ∈ [0, 1] and $\overset{ñ}{\underset{\varphi =1}{\sum }}{♭}_{\varphi }=1$, then


$$\begin{array}{l} \text{2TL}T\text{-SFSSWA}\left(\Upsilon_{1},\Upsilon_{2},\ldots ,\Upsilon_{ñ}\right) \end{array}$$

(5) $$\begin{array}{l} =\left(\begin{array}{c} \Delta \left(‡{\left(1-{\left(\overset{ñ}{\underset{\varphi =1}{\sum }}{♭}_{\varphi }{\left(1-{\left(\frac{\Delta^{-1}\left({ℑ}_{{𝔯}_{\varphi }},R_{\varphi }\right)}{‡}\right)}^{q}\right)}^{k}\right)}^{\frac{1}{k}}\right)}^{\frac{1}{q}}\right),\\ \Delta \left(‡{\left(\overset{ñ}{\underset{\varphi =1}{\sum }}{♭}_{\varphi }{\left(\frac{\Delta^{-1}\left({ℑ}_{{𝔱}_{\varphi }},T_{\varphi }\right)}{‡}\right)}^{qk}\right)}^{\frac{1}{qk}}\right),\\ \Delta \left(‡{\left(\overset{ñ}{\underset{\varphi =1}{\sum }}{♭}_{\varphi }{\left(\frac{\Delta^{-1}\left({ℑ}_{{𝔩}_{\varphi }},L_{\varphi }\right)}{‡}\right)}^{qk}\right)}^{\frac{1}{qk}}\right) \end{array}\right). \end{array}$$

Proof. We prove that the Equation 5 holds by using the mathematical induction method for positive integer ñ.

(a) When ñ = 2, then
$$\begin{array}{l} {♭}_{1}\Upsilon_{1}\\ =\left(\begin{array}{c} \Delta \left(‡{\left(1-{\left(\overset{2}{\underset{\varphi =1}{\sum }}{♭}_{1}{\left(1-{\left(\frac{\Delta^{-1}\left({ℑ}_{{𝔯}_{1}},R_{1}\right)}{‡}\right)}^{q}\right)}^{k}\right)}^{\frac{1}{k}}\right)}^{\frac{1}{q}}\right),\\ \Delta \left(‡{\left(\overset{2}{\underset{\varphi =1}{\sum }}{♭}_{1}{\left(\frac{\Delta^{-1}\left({ℑ}_{{𝔱}_{1}},T_{1}\right)}{‡}\right)}^{qk}\right)}^{\frac{1}{qk}}\right),\\ \Delta \left(‡{\left(\overset{2}{\underset{\varphi =1}{\sum }}{♭}_{1}{\left(\frac{\Delta^{-1}\left({ℑ}_{{𝔩}_{1}},L_{1}\right)}{‡}\right)}^{qk}\right)}^{\frac{1}{qk}}\right) \end{array}\right). \end{array}$$
  Thus, Equation 5 holds for ñ = 2.
(b) Let Equation 5 satisfy with condition $ñ=\tilde{𝔪}$,
$$\begin{array}{l} \text{2TL}T\text{-SFSSWA}\left(\Upsilon_{1},\Upsilon_{2},\ldots ,\Upsilon_{\varphi }\right)\\ =\left(\begin{array}{c} \Delta \left(‡{\left(1-{\left(\overset{\tilde{𝔪}}{\underset{\varphi =1}{\sum }}{♭}_{\varphi }{\left(1-{\left(\frac{\Delta^{-1}\left({ℑ}_{{𝔯}_{\varphi }},R_{\varphi }\right)}{‡}\right)}^{q}\right)}^{k}\right)}^{\frac{1}{k}}\right)}^{\frac{1}{q}}\right),\\ \Delta \left(‡{\left(\overset{\tilde{𝔪}}{\underset{\varphi =1}{\sum }}{♭}_{\varphi }{\left(\frac{\Delta^{-1}\left({ℑ}_{{𝔱}_{\varphi }},T_{\varphi }\right)}{‡}\right)}^{qk}\right)}^{\frac{1}{qk}}\right),\\ \Delta \left(‡{\left(\overset{\tilde{𝔪}}{\underset{\varphi =1}{\sum }}{♭}_{\varphi }{\left(\frac{\Delta^{-1}\left({ℑ}_{{𝔩}_{\varphi }},L_{\varphi }\right)}{‡}\right)}^{qk}\right)}^{\frac{1}{qk}}\right) \end{array}\right). \end{array}$$
  By inductive assumption as $ñ=\tilde{𝔪}+1$, we have
$$\begin{array}{l} \text{2TL}T\text{-SFSSWA}\left(\Upsilon_{1},\Upsilon_{2},\ldots ,\Upsilon_{\tilde{𝔪}},\Upsilon_{\tilde{𝔪}+1}\right)\\ =\text{2TL}T\text{-SFSSWA}\left(\Upsilon_{1},\Upsilon_{2},\ldots ,\Upsilon_{\tilde{𝔪}}\right)⊕{♭}_{\tilde{𝔪}+1}\Upsilon_{\tilde{𝔪}+1} \end{array}$$
$$\begin{array}{l} =\left(\begin{array}{c} \Delta \left(‡{\left(1-{\left(\overset{\tilde{𝔪}}{\underset{\varphi =1}{\sum }}{♭}_{\varphi }{\left(1-{\left(\frac{\Delta^{-1}\left({ℑ}_{{𝔯}_{\varphi }},R_{\varphi }\right)}{‡}\right)}^{q}\right)}^{k}\right)}^{\frac{1}{k}}\right)}^{\frac{1}{q}}\right),\\ \Delta \left(‡{\left(\overset{\tilde{𝔪}}{\underset{\varphi =1}{\sum }}{♭}_{\varphi }{\left(\frac{\Delta^{-1}\left({ℑ}_{{𝔱}_{\varphi }},T_{\varphi }\right)}{‡}\right)}^{qk}\right)}^{\frac{1}{qk}}\right),\\ \Delta \left(‡{\left(\overset{\tilde{𝔪}}{\underset{\varphi =1}{\sum }}{♭}_{\varphi }{\left(\frac{\Delta^{-1}\left({ℑ}_{{𝔩}_{\varphi }},L_{\varphi }\right)}{‡}\right)}^{qk}\right)}^{\frac{1}{qk}}\right) \end{array}\right)\\ ⊕\left(\begin{array}{c} \Delta \left(‡{\left(1-{\left({♭}_{\tilde{𝔪}+1}{\left(1-{\left(\frac{\Delta^{-1}\left({ℑ}_{{𝔯}_{\tilde{𝔪}+1}},R_{\tilde{𝔪}+1}\right)}{‡}\right)}^{q}\right)}^{k}\right)}^{\frac{1}{k}}\right)}^{\frac{1}{q}}\right),\\ \Delta \left(‡{\left({♭}_{\tilde{𝔪}+1}{\left(\frac{\Delta^{-1}\left({ℑ}_{{𝔱}_{\tilde{𝔪}+1}},T_{\tilde{𝔪}+1}\right)}{‡}\right)}^{qk}\right)}^{\frac{1}{qk}}\right),\\ \Delta \left(‡{\left({♭}_{\tilde{𝔪}+1}{\left(\frac{\Delta^{-1}\left({ℑ}_{{𝔩}_{\tilde{𝔪}+1}},L_{\tilde{𝔪}+1}\right)}{‡}\right)}^{qk}\right)}^{\frac{1}{qk}}\right) \end{array}\right). \end{array}$$
$$\begin{array}{l} =\left(\begin{array}{c} \Delta \left(‡{\left(1-{\left(\overset{\tilde{𝔪}+1}{\underset{\varphi =1}{\sum }}{♭}_{\varphi }{\left(1-{\left(\frac{\Delta^{-1}\left({ℑ}_{{𝔯}_{\varphi }},R_{\varphi }\right)}{‡}\right)}^{q}\right)}^{k}\right)}^{\frac{1}{k}}\right)}^{\frac{1}{q}}\right),\\ \Delta \left(‡{\left(\overset{\tilde{𝔪}+1}{\underset{\varphi =1}{\sum }}{♭}_{\varphi }{\left(\frac{\Delta^{-1}\left({ℑ}_{{𝔱}_{\varphi }},T_{\varphi }\right)}{‡}\right)}^{qk}\right)}^{\frac{1}{qk}}\right),\\ \Delta \left(‡{\left(\overset{\tilde{𝔪}+1}{\underset{\varphi =1}{\sum }}{♭}_{\varphi }{\left(\frac{\Delta^{-1}\left({ℑ}_{{𝔩}_{\varphi }},L_{\varphi }\right)}{‡}\right)}^{qk}\right)}^{\frac{1}{qk}}\right) \end{array}\right). \end{array}$$

Based on the provided information, Equation 5 is verified to be valid for the situation where $ñ=\tilde{𝔪}+1$, where ñ and $\tilde{𝔪}$ are positive integers. By employing the mathematical induction method, it can be inferred that Equation 5 remains true for all positive integers ñ ≥ 1.

Theorem 2. Let $\Upsilon_{\varphi }=\left(\left({ℑ}_{{𝔯}_{\varphi }},R_{\varphi }\right),\left({ℑ}_{{𝔱}_{\varphi }},T_{\varphi }\right),\left({ℑ}_{{𝔩}_{\varphi }},L_{\varphi }\right)\right)$ and $\Upsilon_{\varphi }^{\prime }=\left(\left({ℑ}_{{𝔯}_{\varphi }}^{\prime },R_{\varphi }^{\prime }\right),\left({ℑ}_{{𝔱}_{\varphi }}^{\prime },T_{\varphi }^{\prime }\right),\left({ℑ}_{{𝔩}_{\varphi }}^{\prime },L_{\varphi }^{\prime }\right)\right)\left(\varphi =1,2,\ldots ,ñ\right)$ be two sets of 2TL*T*-SFNs; then the 2TL*T*-SFSSWA operator possesses the subsequent properties:

1. (Idempotency) If all $\Upsilon_{\varphi }=\left(\left({ℑ}_{{𝔯}_{\varphi }},R_{\varphi }\right),\left({ℑ}_{{𝔱}_{\varphi }},T_{\varphi }\right)\left({ℑ}_{{𝔩}_{\varphi }},L_{\varphi }\right)\right)\left(\varphi =1,2,\ldots ,ñ\right)$ are equal, for all φ, then
$$\begin{array}{l} \text{2TL}T\text{-SFSSWA}\left(\Upsilon_{1},\Upsilon_{2},\ldots ,\Upsilon_{ñ}\right)=\Upsilon . \end{array}$$
2. (Monotonicity) If $\Upsilon_{\varphi }\leq \Upsilon_{\varphi }^{\prime }$, for all φ, then
$$\begin{array}{l} \text{2TL}T\text{-SFSSWA}\left(\Upsilon_{1},\Upsilon_{2},\ldots ,\Upsilon_{ñ}\right)\\ \leq \text{2TL}T\text{-SFSSWA}\left(\Upsilon_{1}^{\prime },\Upsilon_{2}^{\prime },\ldots ,\Upsilon_{ñ}^{\prime }\right). \end{array}$$
3. (Boundedness) Let $\Upsilon_{\varphi }=\left(\left({ℑ}_{{𝔯}_{\varphi }},R_{\varphi }\right),\left({ℑ}_{{𝔱}_{\varphi }},T_{\varphi }\right)\left({ℑ}_{{𝔩}_{\varphi }},L_{\varphi }\right)\right)\left(\varphi =1,2,\ldots ,ñ\right)$ be an accumulation of 2TL*T*-SFNs, and let $\Upsilon^{-}=\left(\underset{\varphi }{\min}\left({ℑ}_{{𝔯}_{\varphi }},R_{\varphi }\right),\underset{\varphi }{\max}\left({ℑ}_{{𝔱}_{\varphi }},T_{\varphi }\right),\underset{\varphi }{\max}\left({ℑ}_{{𝔩}_{\varphi }},L_{\varphi }\right)\right)$ and $\Upsilon^{+}=\left(\underset{\varphi }{\max}\left({ℑ}_{{𝔯}_{\varphi }},R_{\varphi }\right),\underset{\varphi }{\min}\left({ℑ}_{{𝔱}_{\varphi }},T_{\varphi }\right)\right)$, $\underset{\varphi }{\min}\left({ℑ}_{{𝔩}_{\varphi }},L_{\varphi }\right))$, then
$$\begin{array}{l} \Upsilon^{-}\leq \text{2TL}T\text{-SFSSWA}\left(\Upsilon_{1},\Upsilon_{2},\ldots ,\Upsilon_{ñ}\right)\leq \Upsilon^{+}. \end{array}$$

Definition 12. Let $\Upsilon_{\varphi }=\left(\left({ℑ}_{{𝔯}_{\varphi }},R_{\varphi }\right),\left({ℑ}_{{𝔱}_{\varphi }},T_{\varphi }\right)\left({ℑ}_{{𝔩}_{\varphi }},L_{\varphi }\right)\right)\left(\varphi =1,2,\ldots ,ñ\right)$ be an accumulation of 2TL*T*-SFNs. The 2TL*T*-ROFSSWG operator is a mapping *T*^ñ^ → *T* such that


(6) $$\begin{array}{l} \text{2TL}T\text{-ROFSSWG}\left(\Upsilon_{1},\Upsilon_{2},\ldots ,\Upsilon_{ñ}\right)={⊗}_{\varphi =1}^{ñ}\Upsilon_{\varphi }^{{♭}_{\varphi }}, \end{array}$$

in which *T* is the set of 2TL*T*-SFNs, $♭={\left({♭}_{1},{♭}_{2},\ldots ,{♭}_{ñ}\right)}^{T}$ is the weight vector of Υ_φ_(φ = 1, 2, …, ñ), such that ♭_φ_ ∈ [0, 1] and $\overset{ñ}{\underset{\varphi =1}{\sum }}{♭}_{\varphi }=1.$

Theorem 3. Let $\Upsilon_{\varphi }=\left(\left({ℑ}_{{𝔯}_{\varphi }},R_{\varphi }\right),\left({ℑ}_{{𝔱}_{\varphi }},T_{\varphi }\right)\left({ℑ}_{{𝔩}_{\varphi }},L_{\varphi }\right)\right)\left(\varphi =1,2,\ldots ,ñ\right)$ be an accumulation of 2TL*T*-SFNs with weight vector $♭={\left({♭}_{1},{♭}_{2},\ldots ,{♭}_{ñ}\right)}^{T}$, such that ♭_φ_ ∈ [0, 1] and $\overset{ñ}{\underset{\varphi =1}{\sum }}{♭}_{\varphi }=1$.

The aggregate answer obtained by applying the 2TL*T*-SFSSWG operator remains a 2TL*T*-SFN, and


(7) $$\begin{array}{l} \text{2TL}T\text{-SFSSWG}\left(\Upsilon_{1},\Upsilon_{2},\ldots ,\Upsilon_{ñ}\right)\\ =\left(\begin{array}{c} \Delta \left(‡{\left(\overset{ñ}{\underset{\varphi =1}{\sum }}{♭}_{\varphi }{\left(\frac{\Delta^{-1}\left({ℑ}_{{𝔯}_{\varphi }},R_{\varphi }\right)}{‡}\right)}^{qk}\right)}^{\frac{1}{qk}}\right),\\ \Delta \left(‡{\left(1-{\left(\overset{ñ}{\underset{\varphi =1}{\sum }}{♭}_{\varphi }{\left(1-{\left(\frac{\Delta^{-1}\left({ℑ}_{{𝔱}_{\varphi }},G_{\varphi }\right)}{‡}\right)}^{q}\right)}^{k}\right)}^{\frac{1}{q}}\right)}^{\frac{1}{k}}\right),\\ \Delta \left(‡{\left(1-{\left(\overset{ñ}{\underset{\varphi =1}{\sum }}{♭}_{\varphi }{\left(1-{\left(\frac{\Delta^{-1}\left({ℑ}_{{𝔩}_{\varphi }},L_{\varphi }\right)}{‡}\right)}^{q}\right)}^{k}\right)}^{\frac{1}{q}}\right)}^{\frac{1}{k}}\right) \end{array}\right). \end{array}$$

The arguments are similar to the one used in Theorems 1, 2.

## 5 WASPAS approach based on the 2TL*T*-SFS

MAGDM is a captivating and powerful approach that shines brightly in the realm of decision sciences. Its beauty lies in its ability to harmonize diverse perspectives and preferences within a group, leading to informed and well-rounded decisions. MAGDM gracefully navigates the complex terrain of decision-making by considering multiple criteria, making it a versatile tool for tackling intricate problems across various domains, from business strategy to environmental planning. The elegance of MAGDM stems from its capacity to transform seemingly disparate inputs into a structured framework, facilitating consensus-building and ensuring that the final choice aligns with the collective wisdom of the group. In an era marked by increasing complexity and inter-connectivity, the beauty of MAGDM shines as a beacon of clarity, offering a structured path toward effective and equitable decision-making. The WASPAS approach in MAGDM is a truly remarkable and elegant method that brings a sense of clarity and precision to the often complex task of reaching consensus within a diverse group. Its beauty lies in its ability to harmonize multiple criteria and expert opinions by employing a weighted aggregation technique that considers both the importance of each criterion and the preferences of individual DMs. By effectively balancing the competing interests and perspectives within a group, the WASPAS approach facilitates a fair and structured decision-making process, ensuring that every voice is heard and valued. Its mathematical rigor and intuitive appeal make it a powerful tool for tackling even the most intricate decision problems, making it an invaluable asset in the world of MAGDM.

The concept of a *T*-SFS in MAGDM is a remarkable and powerful tool that enhances the precision and flexibility of decision-making processes. This innovative approach combines the advantages of 2TL representation model, which captures not only the MD but also the NMD and the AD, with the geometric properties of *T*-SFS, which enable the modeling of complex and uncertain information. The beauty of this framework lies in its ability to handle decision problems involving ambiguity and vagueness effectively, making it particularly well-suited for real-world applications where decision criteria are often expressed in qualitative and imprecise terms. By leveraging the synergy of these two mathematical constructs, the 2TL*T*-SFS enriches the MAGDM methodology, providing DMs with a versatile and expressive tool to tackle complex decision scenarios with grace and precision. The SS class of *t*-conorms and *t*-norms in MAGDM is a breathtaking mathematical framework that enchants researchers and practitioners alike with its elegance and versatility. Their beauty lies in their ability to capture complex interdependencies among attributes and criteria in a systematic manner, allowing for more nuanced and flexible modeling of decision processes. As a cornerstone of MAGDM, the SS operations empowers DMs to navigate the intricate landscape of MAGDM problems with grace and precision, making it an indispensable tool in the pursuit of optimal choices in diverse real-world scenarios.

By comprehensive above we construct a novel MAGDM approach based on the 2TL*T*-SF-WASPAS model in which the data is aggregated by a novel 2TL*T*-SFSSWA operator.

**Step 1**. Initiate the alternatives and attributes:
  There is a set of 𝔢 DMs Π = {Π_1_, Π_2_, …, Π_𝔢_} with weights ${♭}^{\prime }={\left({♭}_{1}^{\prime },{♭}_{2}^{\prime },\ldots ,{♭}_{𝔢}^{\prime }\right)}^{T}$ where ♭′ ∈ [0, 1] and $\overset{𝔢}{\underset{\kappa =1}{\sum }}{♭}_{\kappa }^{\prime }=1$ suggests the 𝔓 alternatives ℧ = {℧_1_, ℧_2_, …, ℧_𝔓_} and 𝔉 attributes 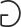 = {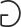
_1_,
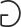
_2_, …,G_𝔉_} with weighs $♭={\left({♭}_{1},{♭}_{2},\ldots ,{♭}_{𝔉}\right)}^{T}$, fulfilling ♭_φ_ ∈ [0, 1], $\overset{𝔉}{\underset{\varphi =1}{\sum }}{♭}_{\varphi }=1$. On the basis of comprehension that is both limited and unclear about alternatives, the DMs give 2TL*T*-SFNs ${ℸ}_{\theta \varphi }^{\kappa }=\left(\left({ℑ}_{{𝔯}_{\theta \varphi }}^{\kappa },R_{\theta \varphi }^{\kappa }\right),\left({ℑ}_{{𝔱}_{\theta \varphi }}^{\kappa },T_{\theta \varphi }^{\kappa }\right),\left({ℑ}_{{𝔩}_{\theta \varphi }}^{\kappa },L_{\theta \varphi }^{\kappa }\right)\right)$ to evaluate their decision concerning various ℧_θ_(θ = 1, 2, …, 𝔓) options relative to the 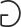
_φ_(φ = 1, 2, …, 𝔉) factor.
**Step 2**. Create the assessment matrix for the 2TL*T*-SFS as follows:
$$X={\left[{ℸ}_{\theta \varphi }^{\kappa }\right]}_{𝔓\times 𝔉}={\left(\left({ℑ}_{{𝔯}_{\theta \varphi }}^{\kappa },R_{\theta \varphi }^{\kappa }\right),\left({ℑ}_{{𝔱}_{\theta \varphi }}^{\kappa },T_{\theta \varphi }^{\kappa }\right),\left({ℑ}_{{𝔩}_{\theta \varphi }}^{\kappa },L_{\theta \varphi }^{\kappa }\right)\right)}_{𝔓\times 𝔉}$$
  where ${ℸ}_{\theta \varphi }^{\kappa }=\left(\left({ℑ}_{{𝔯}_{\theta \varphi }}^{\kappa },R_{\theta \varphi }^{\kappa }\right),\left({ℑ}_{{𝔱}_{\theta \varphi }}^{\kappa },T_{\theta \varphi }^{\kappa }\right),\left({ℑ}_{{𝔩}_{\theta \varphi }}^{\kappa },L_{\theta \varphi }^{\kappa }\right)\right)(\theta =1,2,\ldots ,𝔓,\varphi =1,2,\ldots ,𝔉,$ and κ = 1, 2, …, 𝔢) explains the 2TL*T*-SF detail of options ℧_θ_ on attributes 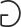
_φ_ by DMs Π_κ_.
**Step 3**. Normalize the decision matrix:
  Equation 8 serves the purpose of standardizing both the benefit index and the cost-type index.
(8) $$\begin{array}{l} {ℸ}_{\theta \varphi }^{\kappa }=\{\begin{array}{c} \left(\left({ℑ}_{{𝔯}_{\theta \varphi }}^{\kappa },R_{\theta \varphi }^{\kappa }\right),\left({ℑ}_{{𝔱}_{\theta \varphi }}^{\kappa },T_{\theta \varphi }^{\kappa }\right),\left({ℑ}_{{𝔩}_{\theta \varphi }}^{\kappa },L_{\theta \varphi }^{\kappa }\right)\right)\in I_{1},\\ \left(\left({ℑ}_{{𝔩}_{\theta \varphi }}^{\kappa },L_{\theta \varphi }^{\kappa }\right),\left({ℑ}_{{𝔱}_{\theta \varphi }}^{\kappa },T_{\theta \varphi }^{\kappa }\right),\left({ℑ}_{{𝔯}_{\theta \varphi }}^{\kappa },R_{\theta \varphi }^{\kappa }\right)\right)\in I_{2} \end{array} \end{array}$$
  *I*_1_ represents the benefit index, while *I*_2_ stands for the cost index.
**Step 4**. Obtain the aggregated 2TL*T*-SF decision matrix to get optimal alternative(s).
(9) $$\begin{array}{l} {ℸ}_{\theta \varphi }=\text{2TL}T\text{-SFSSWA}\left({ℸ}_{\theta \varphi }^{1},{ℸ}_{\theta \varphi }^{2},\ldots ,{ℸ}_{\theta \varphi }^{𝔢}\right), \end{array}$$
**Step 5**. The score function matrix $\left({\overset{⌣}{\Omega }}_{\theta \varphi }\right)$ of the fused decision matrix is calculated by Equation 2 and then the obtained results need to be normalized. Equations 10, 11 are employed for the normalization of the score function matrix, with the former being used for attributes to be maximized, which are benefit-based, and the latter for attributes to be minimized, which are cost-based.
(10) $$\begin{array}{l} {\bar{\Omega }}_{\theta \varphi }=\frac{{\overset{⌣}{\Omega }}_{\theta \varphi }}{\underset{\theta }{\max}{\overset{⌣}{\Omega }}_{\theta \varphi }}, \end{array}$$
(11) $$\begin{array}{l} {\bar{\Omega }}_{\theta \varphi }=\frac{\underset{\theta }{\min}{\overset{⌣}{\Omega }}_{\theta \varphi }}{{\overset{⌣}{\Omega }}_{\theta \varphi }}. \end{array}$$
**Step 6**. Equation 12 computes ${♯}_{\theta }^{\left(1\right)}$, the initial quantity for the overall relative relevance, calculated with the weighted sum model.
(12) $$\begin{array}{l} {♯}_{\theta }^{\left(1\right)}=\overset{ň}{\underset{\varphi =1}{\sum }}{♭}_{\varphi }{\bar{\Omega }}_{\theta \varphi }. \end{array}$$
**Step 7**. Equation 13 computes ${♯}_{\theta }^{\left(2\right)}$, the subsequent overall relative relevance quantity, determined with the help of the weighted product model.
(13) $$\begin{array}{l} {♯}_{\theta }^{\left(2\right)}=\overset{ň}{\underset{\varphi =1}{\prod }}{\left({\bar{\Omega }}_{\theta \varphi }\right)}^{{♭}_{\varphi }}. \end{array}$$
**Step 8**. ♯_θ_ represents the composite optimality value computed using Equation 14. χ denotes the coefficient for composite optimality, and it falls within the range of [0, 1]. When both the weighted sum model and the weighted product model equally influence the composite optimality criteria, χ assumes a value of 0.2.
(14) $$\begin{array}{l} {♯}_{\theta }=\chi {♯}_{\theta }^{\left(1\right)}+\left(1-\chi \right){♯}_{\theta }^{\left(2\right)}. \end{array}$$
**Step 9**. Each alternative is ranked based on its ♯_θ_. The top-ranked alternative is the one that boasts the highest ♯_θ_ value.

## 6 Numerical illustration

To validate our proposed approach, we discuss an illustrative example, perform parameter analysis, and conduct a comparative analysis in this section.

### 6.1 The problem description

Conjunctivitis, also called pink eye in colloquial terms, is a prevalent ocular illness distinguished by the aggravation of the conjunctiva. The conjunctiva is a delicate and transparent layer that coats the lash line's inner surface and envelops the eye's sclera, the white region. This condition can affect people of all ages and is highly contagious. There are several different causes of pink eye, including viral, bacterial, and allergic factors. Buzzing conjunctivitis is frequently characterized by signs like ocular inflammation and the presence of sticky secretion, and itching in the affected eye. It is typically caused by common cold viruses and can spread easily through contact with infected eye secretions or contaminated surfaces. Viral pink eye usually resolves on its own within a week or two without the need for antibiotics. Pathogenic conjunctivitis, however, is attributed to a range of fungal pathogens, including Streptococcus and Staphylococcus. The condition commonly manifests with clinical indicators such as the presence of a dense secretion from the eye, exhibiting a yellow or green hue, accompanied by ocular inflammation and sensations of warmth. The typical course of treatment for fungal conjunctivitis involves the use of antimicrobial eye drops or lotions as recommended by a medical services professional. Allergic conjunctivitis is not infectious and occurs as a result of exposure to allergens like pollen, pet dander, or dust mites. It often presents with itching, redness, and excessive tearing in both eyes. Managing allergic pink eye involves identifying and avoiding allergens, as well as using antihistamine eye drops or other allergy medications as recommended by a healthcare professional.

There are some medications, though, that are not enough to treat pink eye infection. In point of actuality, anybody who makes the attempt to utilize them recklessly runs a chance of causing themselves liability. We evaluate some medications in this article in the hopes that our readers will be wise enough to choose the best medication in order to treat with pink eye infection. For this, there are six medications for the treatment of pink eye infection ℧ = {℧_1_, ℧_2_, …, ℧_6_}, we need to discover the top medication. Each alternative is characterized by a set of attributes denoted as 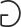 = {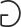
_1_,
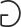
_2_,
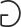
_3_,
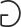
_4_}. These attributes are evaluated with weight vectors, represented as ♭ = (0.2592, 0.2402, 0.2356, 0.2650)^*T*^. Additionally, there are DMs denoted as Π = {Π_1_, Π_2_, Π_3_, Π_4_} who assess these attributes using their respective weight vectors, which are represented as ♭′ = (0.1820, 0.1849, 0.3044, 0.3287)^*T*^. To express the evaluation of the attributes, a Likert-type scale is employed: ℑ^9^={${ℑ}_{0}^{9}$ : extremely bad, ${ℑ}_{1}^{9}$ : very bad, ${ℑ}_{2}^{9}$ : bad, ${ℑ}_{3}^{9}$ : slightly bad, ${ℑ}_{4}^{9}$ : fair, ${ℑ}_{5}^{9}$ : slightly good, ${ℑ}_{6}^{9}$ : good, ${ℑ}_{7}^{9}$ : very good, ${ℑ}_{8}^{9}$ : extremely good}. In this context, four DMs provide their assessments with a high level of competence. Every DM has its own perspective of view regarding recognition of the best medication to treat pink eye infection based on their observations. These medications to treat pink eye infection are:

1. Bleph-10 (℧_1_) (https://www.webmd.com/drugs/2/drug-3707-836/bleph-10-ophthalmic-eye/sulfacetamide-drops-ophthalmic/details);
2. Moxeza (℧_2_) (https://www.rxlist.com/moxeza-drug.htm);
3. Zymar (℧_3_) (https://www.abbvie.ca/content/dam/abbvie-dotcom/ca/en/documents/products/ZYMAR_PI_EN.pdf);
4. Romycin (℧_4_) (https://www.webmd.com/drugs/2/drug-13474/romycin-ophthalmic-eye/details);
5. Polytrim (℧_5_) (https://www.webmd.com/drugs/2/drug-17/polytrim-ophthalmic-eye/details);
6. Bacticin (℧_6_) (https://www.webmd.com/drugs/2/drug-2052/bacitracin-polymyxin-b-ophthalmic-eye/details).

**Figure 7:**
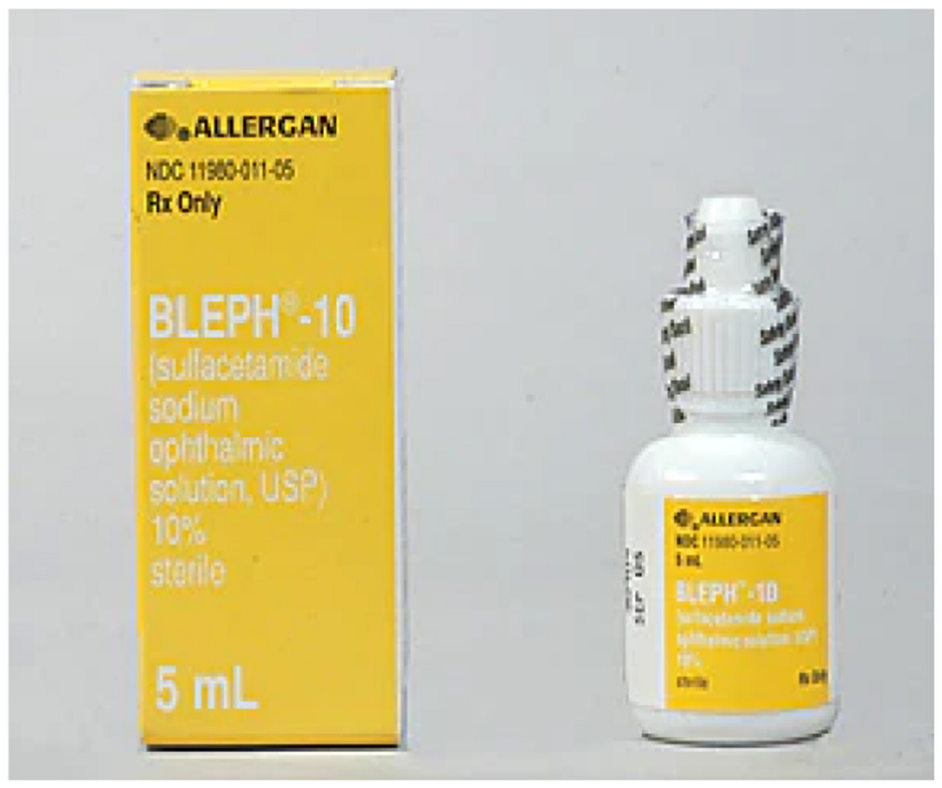
Bleph-10 medication. Reproduced from WedMD, LLC, https://www.webmd.com/drugs/2/drug-4184-836/sulfacetamide-sodium-ophthalmic-eye/sulfacetamide-drops-ophthalmic/details.

A detailed description of six pink eye infection medications including definition, uses, procedure for usage, side effects, and precautions is given as follows:

1. **Bleph-10:** Bleph-10 is a pharmaceutical medication frequently recommended for the therapeutic management of conjunctivitis, colloquially referred to as pink eye. The eye drop solution comprises sulfacetamide sodium, an antibiotic agent that effectively addresses ocular bacterial infections. Conjunctivitis, also known as pink eye, is an infectious ailment that manifests as ocular redness, pruritus, discharge, and ocular discomfort. There are multiple causative factors that can contribute to this condition, encompassing bacteria, viruses, and allergies. Bleph-10 is a pharmaceutical product that has been carefully formulated to selectively target and eradicate bacterial infections, hence rendering it a suitable and efficacious option for the treatment of bacterial conjunctivitis. When using Bleph-10, it is crucial to follow the recommended dosage and application guidelines given by a healthcare professional in order to maximize results and reduce potential side effects (see Figure 7: https://www.webmd.com/drugs/2/drug-3707-836/bleph-10-ophthalmic-eye/sulfacetamide-drops-ophthalmic/details).

**Uses:**

The indicated purpose of this medication is to address ocular bacterial infections, specifically conjunctivitis. This medication is classified as a member of the sulfa antibiotic pharmacological class. The mechanism of action of sulfacetamide involves the inhibition of bacterial growth. This drug is specifically indicated for the treatment of bacterial eye infections. This treatment modality is not efficacious for various other forms of ocular infections. The unnecessary utilization or improper administration of any antibiotic can result in a decline in its efficacy.

**Procedure for usage:**

Prior to utilization, visually inspect the product for any signs of discoloration. In the event of discoloration, it is advised to refrain from using the liquid. Prior to applying eye drops, it is essential to cleanse your hands thoroughly. In order to prevent contamination, it is imperative to refrain from making contact with the dropper tip or allowing it to come into contact with the eye or any other surface. Restricted to visual perception exclusively. It is advised against ingesting or administering by injection. Do not wear contact lenses when using this drug. Sterilize contact lenses according to the manufacturer's guidelines, then check with a doctor before using them again. To create a pouch, one should tilt their head backwards, direct their gaze upwards, and then draw down the lower eyelid. Position the dropper in close proximity to the ocular region, and administer a single droplet into the conjunctival sac in accordance with the instructions provided by a medical professional. Direct your gaze downward, gradually close your eyelids, and position one digit in proximity to the inner corner of your eye, adjacent to the nasal area. Prior to opening your eyes, it is recommended to exert a modest amount of pressure for a duration of 1 to 2 minutes. It is advisable to refrain from blinking excessively or engaging in eye rubbing. In the event that it is specified or if the prescribed dosage exceeds a single drop, proceed to replicate these procedures for another eye. It is advisable to allow a few minutes for one's eyesight to fully recover preceding engaging in activities such as transportation or manipulating machines. It is advised to refrain from rinsing the dropper. It is recommended to replenish the dropper cap following every application.

**Side effects:**

The Bleph-10 medication has the following potential side effects:

- Eye stinging/burning/redness and temporary blurred vision may occur;
- Fungal infections;
- Worsening eye symptoms (such as pain, swelling, thick discharge or pus);
- Aching/swollen joints;
- Rash on nose and cheeks;
- Signs of infection (such as sore throat that does not go away, fever);
- Signs of anemia (such as unusual tiredness/weakness, rapid breathing, fast heartbeat);
- Unusual bleeding/bruising;
- Signs of liver problems (such as nausea that does not stop, dark urine, yellowing eyes/skin, stomach/abdominal pain, vomiting);
- Mouth sores.

**Precautions:**

Prior to utilizing sulfacetamide, it is imperative to inform the healthcare provider or pharmacist about any potential hypersensitivity to this medication, other sulfa antibiotics (e.g., sulfamethoxazole), or any other known allergies. The presence of inactive substances in this product has the potential to elicit allergic responses or other adverse effects. For further information, it is advisable to consult with the pharmacist. Prior to utilizing this medication, it is imperative to disclose one's medical history, particularly with regards to the use of contact lenses, to a healthcare professional or pharmacist. Following the administration of this medication, there is a possibility of experiencing a transient period of visual blurring. It is advised to abstain from operating vehicles, utilizing machines, or engaging in any task that necessitates optimal visual acuity. It is vital to inform the medical practitioner, whether a doctor or dentist, about any substances utilized, encompassing prescribed medications, over-the-counter prescriptions, and herbal remedies, prior to undergoing any surgical procedure. During gestation, it is advisable to administer this drug solely in cases where it is deemed necessary. It is advisable to engage in a comprehensive discussion with the medical practitioner regarding the potential hazards and advantages associated with a certain course of action.

**Figure 8:**
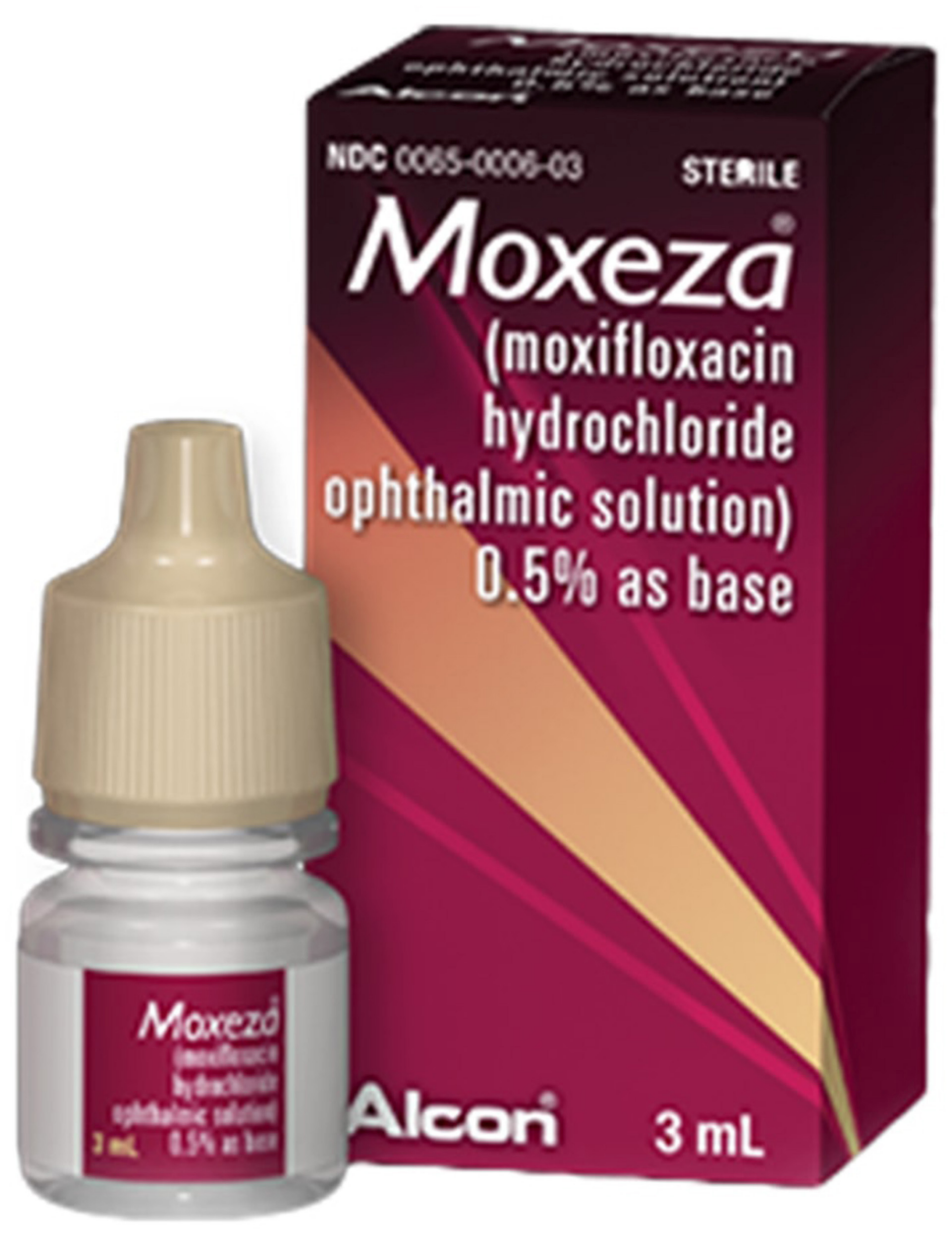
Moxeza medication. Reproduced from Beye, LLC, https://www.beye.com/product/moxeza.

2. **Moxeza:** Moxeza is a frequently prescribed medication utilized in the management of conjunctivitis, often referred to as pink eye, an infectious ocular infection. The ocular solution in question comprises moxifloxacin, a strong antibiotic known for its efficacy against a diverse array of bacteria that are causative agents of bacterial conjunctivitis. When administered according to the instructions of a healthcare practitioner, Moxeza functions by impeding the proliferation of bacteria and diminishing ocular irritation. The medication is conveniently accessible in the form of ocular drops, facilitating ease of application and delivering prompt alleviation from the symptoms of ocular conjunctivitis, including discomfort and ocular erythema. Moxeza is commonly prescribed for a brief period, typically spanning 7–10 days, and the frequency of dosage is decided based on the severity of the illness (see Figure 8: https://www.beye.com/product/moxeza).

**Uses:**

This medicine, a quinolone antibiotic, is used to treat eye diseases.

**Procedure for usage:**

For optimal outcomes, it is recommended to adhere strictly to the provided instructions for the entire duration, as advised. Premature discontinuation of this medicine may lead to a recurrence of the infection. Before using eye drops, it is important to first cleanse your hands. In order to prevent contamination, it is imperative to ensure that the dropper tip does not come into contact with any surface. In order to create a pouch, one should tilt their head backwards, direct their gaze upwards, and then draw down the lower eyelid. Position the dropper in direct alignment with the eye and administer the designated quantity of drops as advised. Direct your gaze downward and proceed to softly close your eyes for a duration of 1 to 2 minutes. Position a single digit in proximity to the ocular region adjacent to the nasal area, then exert a mild amount of force.

**Side effects:**

The Moxeza medication has the following potential side effects:

- Blurred vision;
- Eye pain/dryness/redness/itchiness;
- Swelling of the eye;
- Rash;
- Itching/swelling (especially of the face/tongue/throat);
- Dizziness;
- Trouble breathing.

**Precautions:**

Prior to initiating the administration of this medication, it is imperative to inform your healthcare provider or pharmacist of any potential adverse reactions to the medication itself, quinolone antibiotics (such as ciprofloxacin), or any other known allergies. The presence of inactive substances in this product has the potential to elicit allergic responses or other adverse effects. For further information, it is advisable to consult with the pharmacist. Prior to initiating the administration of this medication, it is imperative to disclose pertinent medical history to the attending physician or pharmacist, with particular emphasis on the utilization of contact lenses. Following the administration of this medication, there is a possibility of experiencing a temporary occurrence of blurred or unstable eyesight. It is advisable to refrain from blinking and avoid rubbing the eye. It is advised to refrain from rinsing the dropper. It is advisable to observe a minimum interval of five minutes before administering other drugs subsequent to the use of alternative eye drops. It is advised to refrain from wearing contact lenses during the administration of this medication. It is imperative to adhere to the sterilization instructions provided by the contact lens manufacturer and consult a medical professional prior to their usage. It is advised to promptly notify the physician if there is no amelioration of the ailment during a period of seven days. It is advised to refrain from operating vehicles, utilizing machinery, or engaging in any tasks that necessitate optimal visual acuity. The administration of this drug over an extended or recurring period may lead to the development of a secondary infection. During gestation, it is advisable to utilize this drug solely in instances where it is deemed necessary. It is advisable to engage in a comprehensive discussion with the doctor regarding the potential dangers and advantages associated with the subject matter.

3. **Zymar:** Zymar is a popular prescribed medication used to treat conjunctivitis, sometimes known as pink eye, when the infection is bacterial. The antibiotic gatifloxacin, which is found in this ophthalmic solution, is a member of the fluoroquinolone family. Zymar relieves symptoms by stopping the spread of bacteria in the infected eye, which ultimately kills off the infection. It is prescribed for a short period of time, usually 5–7 days, depending on the severity of the infection, and it is available in the form of eye drops, making it easier to apply straight to the affected eye. If your doctor has prescribed Zymar to treat pink eye, be sure to use it exactly as directed. Be sure to use clean hands before administering the eye drops, and stay away from touching the dropper tip (see https://www.practo.com/medicine-info/zymar-03-drops-19162).

**Uses:**

Zymar eye drops are typically prescribed for a duration of one week. During the initial two days, it is advised to administer one drop every two hours in the affected eye(s) while awake, with a maximum of eight times per day. From days three to seven, one drop should be applied four times a day while awake. It is important to distribute the doses evenly throughout the day. Various factors, such as body weight, other medical conditions, and concurrent medications, can influence the required dosage of this medication. If a healthcare professional has recommended a different dosage regimen, it is crucial to consult them before altering the administration method. It is advisable to seek guidance from a doctor or pharmacist regarding the appropriate technique for applying eye drops. The safety and efficacy of using Zymar eye drops in children under one year of age have not been established. However, Zymar ophthalmic solution has been utilized for treating conjunctivitis in children aged one to twelve years.

**Procedure for usage:**

Preparing to take medication requires washing your hands. Lean back and gaze up at the sky. To make a little pouch under your eye, draw down your lower lid gently. Invert the container and carefully place one drop into the eye. Relax your eyelids and close them for thirty seconds.

**Side effects:**

The Zymar medication has the following potential side effects:

- Decreased vision;
- Dizziness;
- Dry eye;
- Eye irritation;
- Eye pain;
- Nausea;
- Runny nose;
- Sneezing;
- Sore throat;
- Swelling and redness of the eyelid;
- Tearing or eye discharge;
- Unusual aftertaste.

Most of the below-listed side effects are uncommon, but they could become severe if somebody did not seek medical assistance.

- Blurred vision;
- Bacterial resistance;
- Pain or swelling of a tendon;
- Sensitivity to light;
- Spots on the cornea;
- Swelling of the cornea;
- Other disorders of the area around the cornea.

**Precautions:**

Prior to initiating pharmaceutical usage, it is imperative to duly apprise the attending physician of any prevailing medical problems or allergies, disclose the ongoing consumption of any medications, ascertain the pregnancy or breastfeeding status of any female individuals, and provide any other pertinent health-related information. Several factors can potentially influence the appropriate use of this drug. It is advisable to refrain from using contact lenses while exhibiting indications and manifestations of bacterial conjunctivitis. Additionally, this drug is formulated with a preservative that has the potential to cause discoloration of soft contact lenses. The administration of this medicine during pregnancy is contraindicated unless the potential advantages outweigh the potential hazards. The passage of Zymar into breast milk remains uncertain. The administration of this medication has the potential to induce severe allergic reactions, such as anaphylaxis. Anaphylaxis is a potentially fatal condition that necessitates prompt medical intervention. It is imperative to promptly seek medical attention in the event of experiencing symptoms such as a rash, pruritus, erythema, or edema in the vicinity of the eye or eyelid, respiratory difficulties, dysphagia, or any kind of edema affecting the hands, face, or oral cavity during the course of this medication. This medication has the potential to cause severe skin responses. It is advisable to promptly consult a medical professional if experiencing symptoms such as blistering, peeling, or detachment of the skin, the presence of red skin lesions, severe acne or skin rash, the development of sores or ulcers on the skin, or the occurrence of fever or chills throughout the course of medication.

4. **Romycin:** Romycin, or erythromycin ophthalmic ointment, is a frequently prescribed pharmaceutical agent utilized in the management of conjunctivitis, a prevalent and communicable ocular illness. The ointment in question comprises erythromycin, a type of macrolide antibiotic that demonstrates efficacy against a wide range of microorganisms known to induce conjunctivitis. When administered in a topical manner to the eye that is impacted, Romycin functions by impeding the proliferation and replication of bacteria that are accountable for the illness. The utilization of this treatment is particularly advantageous in instances of conjunctivitis resulting from bacterial etiology, as it effectively alleviates symptoms such as ocular redness, pruritus, and discharge. Romycin is commercially accessible in the form of an ophthalmic ointment, facilitating its administration to individuals of all age groups, including children and adults. The standard prescribed dosage is the application of a slender strip onto the lower eyelid, with a frequency of two to four times per day. However, it is imperative to seek guidance from a healthcare practitioner prior to utilizing Romycin in order to ascertain the accurate diagnostic and treatment regimen for conjunctivitis, as not all instances may necessitate antibiotic intervention (see https://www.bernell.com/product/AK45O3DS/$Index_E$).

**Uses:**

This medication is prescribed for the treatment of conjunctivitis and other similar eye infections. It's also given to babies to stop them from getting an infection in their eyes. It is an antibiotic in the macrolide family. Romycin inhibits bacterial growth, hence its efficacy. Bacterial eye infections are the only ones that this drug can treat. Other forms of eye infection will not be helped by this.

**Procedure for usage:**

You should clean your hands before applying eye ointment. Avoid getting any part of the tube's tip near your eye, eyelid, or any other part of your body to keep from spreading germs. Only use it on the eyes. Avoid ingesting or injecting. Do not use this medication if you plan on wearing contact lenses. Clean and disinfect your contact lenses as directed by the manufacturer, and have your eyes checked before reusing them. Tilt your head back, raise your brow, and pull down your lower eyelid to create a pouch for eye drops. After consulting with your doctor, insert an ointment strip of about half an inch (1 centimeter) into the pouch. To evenly distribute the drug, softly close the eye and gently roll the eyeball in all directions. Don't rub your eyes or blink. If instructed to do so, repeat the process with your other eye. Remove any excess medication from the ointment tube's tip with a clean tissue before recapping. Don't go behind the wheel or operate heavy machinery for a while until you can see clearly. Medical condition and treatment outcome determine the dosage. Do not use more regularly or raise the dosage without consulting your doctor beforehand. Wait at least 5 minutes between using different eye treatments (drops, ointments, etc.). Eye ointments won't penetrate the eye, so use eye drops first. Take this drug as prescribed to maximize its effectiveness. It will be easier to recall if you use it at the same time every day. Keep using it for the whole amount of time recommended. A recurrence of the illness is possible if the medicine is stopped too soon and the germs are given time to multiply.

**Side effects:**

The Romycin medication has the following potential side effects:

- Eye stinging/burning/redness or temporary blurred vision;
- Itching/swelling (especially of the face/tongue/throat);
- Severe dizziness;
- New or worsening eye symptoms (such as pain, swelling, thick discharge or pus);
- Trouble breathing.

**Precautions:**

If you have an allergy to Romycin or any other macrolide antibiotics (including clarithromycin), you should talk to your doctor or pharmacist before using this medication. Allergies and other reactions to the product's inactive components are possible. For further information, consult your local pharmacy. Tell your doctor or pharmacist everything about your medical history before starting this medicine. Applying this medication can cause momentary blurring of eyesight. Don't operate any vehicles or heavy machinery, and stay away from anything that can blur your vision. Tell your surgeon about any and all medications you're taking, whether they're prescribed, over-the-counter, or herbal, before your procedure. This drug should be used during pregnancy only when absolutely necessary. Get your doctor's opinion on the pros and cons. Romycin's potential to enter breast milk after being used as an eye ointment is not known. A nursing infant is unlikely to be harmed. Before starting to breastfeed, you should see a doctor.

**Figure 9:**
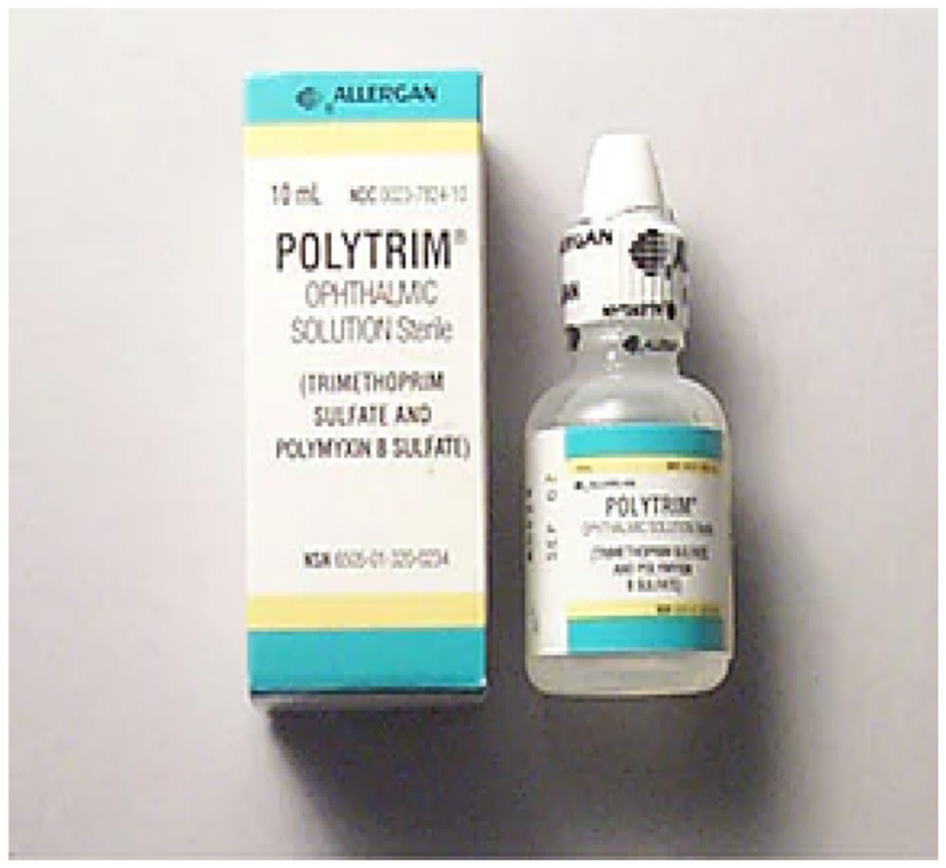
Polytrim medication. Reproduced from WebMD, LLC, https://www.webmd.com/drugs/2/drug-3408/polymyxin-b-sulfate-trimethoprim-ophthalmic-eye/details.

5. **Polytrim:** Polytrim is a medication that is frequently recommended for the management of conjunctivitis, colloquially referred to as pink eye, particularly in cases where the etiology is considered to be bacterial in nature. Conjunctivitis, also known as pink eye, is an extremely transmissible ailment characterized by ocular redness, pruritus, lacrimation, and ocular discharge. Polytrim is a medication that comprises two bioactive components, namely polymyxin B sulfate and trimethoprim. Polymyxin B is an antimicrobial agent that exhibits bactericidal activity against a diverse range of bacterial species, whereas trimethoprim exerts bacteriostatic effects by disrupting the bacterial protein synthesis required for their vital functions. The efficacy of Polytrim in treating bacterial conjunctivitis is attributed to its ability to target a wide range of germs, thereby making it effective against a broad spectrum of often implicated bacterial strains. Adherence to the doctor's recommendations and completion of the entire treatment regimen, particularly in cases where symptoms ameliorate prior to its conclusion, are imperative (see Figure 9: https://www.webmd.com/drugs/2/drug-17/polytrim-ophthalmic-eye/details).

**Uses:**

Bacterial infections of the eye (blepharitis, conjunctivitis) are treated with this medication. There are two antibiotics in it. Polymyxin B eliminates microorganisms, hence its efficacy. Inhibiting further bacterial development is what makes polytrim effective.

**Procedure for usage:**

Before you put in eye drops, you should clean your hands. The dropper tip should not come into contact with the eye or any other surface to prevent the spread of infection. Only for use in the eyes. Avoid ingesting or injecting. Do not use this medication if you plan on wearing contact lenses. Clean and disinfect your contact lenses as directed by the manufacturer, and have your eyes checked before reusing them. Look up and backward while pulling down on the lower eyelid to create a pouch. Place one drop into the pouch by holding the dropper over the eye, as advised by your doctor. Put one finger at the inside corner of your eye (close to the nose) and look down. After one or two minutes of light pressure, you can safely open your eyes. Because of this, the medicine won't leak out. Don't wipe your eyes or blink too much. If more than one drop is required, follow the instructions again. If you were instructed to put the medication in both eyes, do the same thing again. Don't go behind the wheel or operate heavy machinery for a while until you can see clearly. The dropper must not be cleaned. After use, always re-cap the dropper. Condition and treatment response are taken into account while determining dosage. It's possible that your doctor will have you take this drug more frequently at first and then less frequently as the infection clears up. Listen to the advice of your medical professional.

**Side effects:**

The Polytrim medication has the following potential side effects:

- Temporary eye stinging/burning/redness/itchiness or temporary blurred vision;
- Fungal infections;
- Eye symptoms (such as pain, swelling, thick discharge or pus);
- Rash;
- Itching/swelling (especially of the eyes/face/tongue/throat);
- Severe dizziness;
- Trouble breathing.

**Precautions**

Tell your doctor or pharmacist if you are allergic to polymyxin B/trimethoprim or any other medications. Allergies and other reactions to the product's inactive components are possible. For further information, consult your local pharmacy. Tell your doctor or pharmacist everything about your medical history before starting this medicine. Applying this medication can cause momentary blurring of eyesight. Don't operate any vehicles or heavy machinery, and stay away from anything that can blur your vision. Tell your surgeon about any and all medications you're taking, whether they're prescribed, over-the-counter, or herbal, before your procedure. This medication should be used during pregnancy only when absolutely necessary.

6. **Bacticin:** Bacticin is a common medication used to treat conjunctivitis, sometimes known as pink eye. One of the most common causes of conjunctivitis is an infection of the eye, and this drug is effective against germs that cause this illness. Synergistically eliminating the infecting bacteria, bacitracin contains a mixture of antibiotics (usually neomycin and polymyxin B). These antibiotics successfully block bacterial development, stopping infection from worsening and alleviating eye irritation. Bacticin can be applied topically to the affected eye in the form of eye drops or ointment for fast, targeted relief. Using Bacticin in accordance with a doctor's instructions can lessen the redness, swelling, discharge, and discomfort related to bacterial conjunctivitis (see https://www.stagmedical.com/products/bacitacin-zinc-polymyxin-b-sulfate-ointment-3-5g-bausch-lomb).

**Uses:**

Infections of the eye can be treated with this medicine. Antibiotics such as bacitracin and polymyxin are included in this medication. Bacterial eye infections are the only ones that this drug can treat. It is not effective against fungal, viral, or mycobacterial infections of the eye.

**Procedure for usage:**

As prescribed, this medication is topically given to the affected eye(s). Medical condition and treatment outcome determine the dosage. Do not use this medication if you wear contact lenses. Clean and disinfect your contact lenses as directed by the manufacturer, and have your eyes checked before reusing them. Before putting on the eye ointment, make sure your hands are clean. Take care not to contaminate the tube by touching the tip or allowing it to come into contact with the eye or any other surface. Look up and backward while pulling down on the lower eyelid to create a pouch. Squeeze a half-inch (1.5-centimeter) length of ointment from the tube and place it in the bag. When using eye drops, it is best to close the eye and roll the eyeball in all directions. Don't rub your eyes or blink. If instructed to do so, repeat the process with your other eye. Remove any excess medication from the ointment tube's tip with a clean tissue before recapping. Use it as your doctor prescribes. If you're already using eye drops or another type of ointment, give those a few minutes to take effect before using this ointment. You should use eye drops before applying eye ointments so that the drops can enter the eye. If you plan on using another eye ointment, wait at least 10 min after using this prescription. Regular dosing is required for the maximum efficacy of this drug. Maintain a daily routine with it. Even if your symptoms have subsided after a few days, it is still important to take this medication for the complete duration of treatment. If you stop taking your medication too soon, the bacteria in your body may adapt, and you could end up with the same illness all over again. If your eye symptoms (discharge, swelling, redness, and pain) don't resolve after a few days, or if they worsen, make an appointment with your doctor immediately.

**Side effects:**

The Bacticin medication has the following potential side effects:

- Stinging/burning of the eyes for 1–2 min or temporary blurred vision;
- Rash;
- Itching/swelling (especially of the face/tongue/throat/eye/eyelid);
- Severe dizziness;
- Trouble breathing.

**Precautions:**

Tell your doctor or pharmacist if you are allergic to bacitracin, polymyxin, or any other part of this drug. Allergies and other reactions to the product's inactive components are possible. For further information, consult your local pharmacy. You should discuss your medical history with your doctor or pharmacist before starting this drug. Applying this medication can cause momentary blurring of eyesight. Don't operate any vehicles or heavy machinery, and stay away from anything that can blur your vision. This medication should be used during pregnancy only when absolutely necessary.

The 2TL*T*-SFNs provided by four DMs is given in Table 1.

**Table 1:** The evaluation matrix with 2TL*T*-SFNs.

**DMs**	**Medications**	**ℵ_1_**	**ℵ_2_**	**ℵ_3_**	**ℵ_4_**
	℧_1_	((ℑ_3_, 0), (ℑ_1_, 0), (ℑ_4_, 0))	((ℑ_3_, 0), (ℑ_2_, 0), (ℑ_5_, 0))	((ℑ_3_, 0), (ℑ_3_, 0), (ℑ_6_, 0))	((ℑ_4_, 0), (ℑ_1_, 0), (ℑ_7_, 0))
	℧_2_	((ℑ_4_, 0), (ℑ_1_, 0), (ℑ_4_, 0))	((ℑ_4_, 0), (ℑ_2_, 0), (ℑ_4_, 0))	((ℑ_4_, 0), (ℑ_3_, 0), (ℑ_4_, 0))	((ℑ_3_, 0), (ℑ_2_, 0), (ℑ_4_, 0))
Π_1_	℧_3_	((ℑ_4_, 0), (ℑ_3_, 0), (ℑ_4_, 0))	((ℑ_5_, 0), (ℑ_2_, 0), (ℑ_6_, 0))	((ℑ_7_, 0), (ℑ_1_, 0), (ℑ_5_, 0))	((ℑ_4_, 0), (ℑ_4_, 0), (ℑ_5_, 0))
	℧_4_	((ℑ_5_, 0), (ℑ_3_, 0), (ℑ_4_, 0))	((ℑ_4_, 0), (ℑ_3_, 0), (ℑ_5_, 0))	((ℑ_3_, 0), (ℑ_2_, 0), (ℑ_6_, 0))	((ℑ_7_, 0), (ℑ_1_, 0), (ℑ_7_, 0))
	℧_5_	((ℑ_6_, 0), (ℑ_1_, 0), (ℑ_2_, 0))	((ℑ_3_, 0), (ℑ_3_, 0), (ℑ_6_, 0))	((ℑ_4_, 0), (ℑ_2_, 0), (ℑ_5_, 0))	((ℑ_2_, 0), (ℑ_1_, 0), (ℑ_7_, 0))
v	℧_6_	((ℑ_3_, 0), (ℑ_1_, 0), (ℑ_7_, 0))	((ℑ_5_, 0), (ℑ_2_, 0), (ℑ_4_, 0))	((ℑ_5_, 0), (ℑ_2_, 0), (ℑ_6_, 0))	((ℑ_3_, 0), (ℑ_3_, 0), (ℑ_5_, 0))
	℧_1_	((ℑ_2_, 0), (ℑ_2_, 0), (ℑ_5_, 0))	((ℑ_3_, 0), (ℑ_2_, 0), (ℑ_6_, 0))	((ℑ_4_, 0), (ℑ_2_, 0), (ℑ_5_, 0))	((ℑ_4_, 0), (ℑ_4_, 0), (ℑ_4_, 0))
	℧_2_	((ℑ_4_, 0), (ℑ_3_, 0), (ℑ_4_, 0))	((ℑ_5_, 0), (ℑ_3_, 0), (ℑ_5_, 0))	((ℑ_3_, 0), (ℑ_3_, 0), (ℑ_6_, 0))	((ℑ_5_, 0), (ℑ_2_, 0), (ℑ_7_, 0))
Π_2_	℧_3_	((ℑ_3_, 0), (ℑ_2_, 0), (ℑ_5_, 0))	((ℑ_4_, 0), (ℑ_3_, 0), (ℑ_7_, 0))	((ℑ_2_, 0), (ℑ_1_, 0), (ℑ_6_, 0))	((ℑ_4_, 0), (ℑ_2_, 0), (ℑ_6_, 0))
	℧_4_	((ℑ_5_, 0), (ℑ_3_, 0), (ℑ_7_, 0))	((ℑ_4_, 0), (ℑ_3_, 0), (ℑ_5_, 0))	((ℑ_4_, 0), (ℑ_4_, 0), (ℑ_5_, 0))	((ℑ_5_, 0), (ℑ_2_, 0), (ℑ_7_, 0))
	℧_5_	((ℑ_5_, 0), (ℑ_2_, 0), (ℑ_7_, 0))	((ℑ_6_, 0), (ℑ_2_, 0), (ℑ_6_, 0))	((ℑ_4_, 0), (ℑ_2_, 0), (ℑ_7_, 0))	((ℑ_4_, 0), (ℑ_1_, 0), (ℑ_4_, 0))
	℧_6_	((ℑ_3_, 0), (ℑ_1_, 0), (ℑ_7_, 0))	((ℑ_5_, 0), (ℑ_3_, 0), (ℑ_7_, 0))	((ℑ_7_, 0), (ℑ_1_, 0), (ℑ_6_, 0))	((ℑ_4_, 0), (ℑ_3_, 0), (ℑ_7_, 0))
	℧_1_	((ℑ_6_, 0), (ℑ_2_, 0), (ℑ_6_, 0))	((ℑ_4_, 0), (ℑ_3_, 0), (ℑ_7_, 0))	((ℑ_5_, 0), (ℑ_2_, 0), (ℑ_7_, 0))	((ℑ_2_, 0), (ℑ_1_, 0), (ℑ_6_, 0))
	℧_2_	((ℑ_5_, 0), (ℑ_1_, 0), (ℑ_5_, 0))	((ℑ_7_, 0), (ℑ_1_, 0), (ℑ_6_, 0))	((ℑ_2_, 0), (ℑ_1_, 0), (ℑ_5_, 0))	((ℑ_4_, 0), (ℑ_4_, 0), (ℑ_7_, 0))
Π_3_	℧_3_	((ℑ_7_, 0), (ℑ_1_, 0), (ℑ_7_, 0))	((ℑ_6_, 0), (ℑ_1_, 0), (ℑ_4_, 0))	((ℑ_4_, 0), (ℑ_3_, 0), (ℑ_7_, 0))	((ℑ_3_, 0), (ℑ_2_, 0), (ℑ_5_, 0))
	℧_4_	((ℑ_7_, 0), (ℑ_1_, 0), (ℑ_6_, 0))	((ℑ_6_, 0), (ℑ_1_, 0), (ℑ_5_, 0))	((ℑ_5_, 0), (ℑ_2_, 0), (ℑ_5_, 0))	((ℑ_7_, 0), (ℑ_1_, 0), (ℑ_5_, 0))
	℧_5_	((ℑ_4_, 0), (ℑ_3_, 0), (ℑ_7_, 0))	((ℑ_5_, 0), (ℑ_1_, 0), (ℑ_6_, 0))	((ℑ_5_, 0), (ℑ_2_, 0), (ℑ_5_, 0))	((ℑ_4_, 0), (ℑ_3_, 0), (ℑ_4_, 0))
	℧_6_	((ℑ_3_, 0), (ℑ_2_, 0), (ℑ_7_, 0))	((ℑ_5_, 0), (ℑ_2_, 0), (ℑ_7_, 0))	((ℑ_7_, 0), (ℑ_1_, 0), (ℑ_7_, 0))	((ℑ_4_, 0), (ℑ_4_, 0), (ℑ_5_, 0))
	℧_1_	((ℑ_6_, 0), (ℑ_1_, 0), (ℑ_7_, 0))	((ℑ_7_, 0), (ℑ_1_, 0), (ℑ_6_, 0))	((ℑ_6_, 0), (ℑ_2_, 0), (ℑ_7_, 0))	((ℑ_5_, 0), (ℑ_1_, 0), (ℑ_6_, 0))
	℧_2_	((ℑ_4_, 0), (ℑ_3_, 0), (ℑ_6_, 0))	((ℑ_5_, 0), (ℑ_2_, 0), (ℑ_7_, 0))	((ℑ_5_, 0), (ℑ_2_, 0), (ℑ_7_, 0))	((ℑ_4_, 0), (ℑ_2_, 0), (ℑ_6_, 0))
Π_4_	℧_3_	((ℑ_4_, 0), (ℑ_4_, 0), (ℑ_5_, 0))	((ℑ_6_, 0), (ℑ_2_, 0), (ℑ_6_, 0))	((ℑ_3_, 0), (ℑ_1_, 0), (ℑ_7_, 0))	((ℑ_4_, 0), (ℑ_1_, 0), (ℑ_5_, 0))
	℧_4_	((ℑ_7_, 0), (ℑ_1_, 0), (ℑ_7_, 0))	((ℑ_6_, 0), (ℑ_2_, 0), (ℑ_7_, 0))	((ℑ_5_, 0), (ℑ_3_, 0), (ℑ_4_, 0))	((ℑ_4_, 0), (ℑ_3_, 0), (ℑ_4_, 0))
	℧_5_	((ℑ_3_, 0), (ℑ_1_, 0), (ℑ_4_, 0))	((ℑ_5_, 0), (ℑ_2_, 0), (ℑ_7_, 0))	((ℑ_5_, 0), (ℑ_1_, 0), (ℑ_6_, 0))	((ℑ_4_, 0), (ℑ_4_, 0), (ℑ_5_, 0))
	℧_6_	((ℑ_3_, 0), (ℑ_1_, 0), (ℑ_7_, 0))	((ℑ_5_, 0), (ℑ_2_, 0), (ℑ_4_, 0))	((ℑ_7_, 0), (ℑ_1_, 0), (ℑ_6_, 0))	((ℑ_5_, 0), (ℑ_2_, 0), (ℑ_5_, 0))

### 6.2 The results of a case study

The 2TL*T*-SF-WASPAS approach based on the 2TL*T*-SFSSWA operator as described in Section 5 is used in this subsection to illustrate the assessment procedure for evaluating the medications to treat pink eye infection.

**Step 1**. Initiate the alternatives and attributes:
  A group of four DMs Π = {Π_1_, Π_2_, Π_3_, Π_4_} with set of DMs weights ♭′ = (0.1820, 0.1849, 0.3044, 0.3287)^*T*^, decides the six alternatives ℧ = {℧_1_, ℧_2_, …, ℧_6_} and four attributes 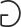 = {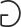
_1_,
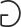
_2_,
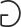
_3_,
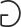
_4_} with weights ♭ = (0.2592, 0.2402, 0.2356, 0.2650)^*T*^. Based on incomplete and ambiguous knowledge about alternatives, the DMs give 2TL*T*-SFNs ${ℸ}_{\theta \varphi }^{\kappa }=\left(\left({ℑ}_{{𝔯}_{\theta \varphi }}^{\kappa },R_{\theta \varphi }^{\kappa }\right),\left({ℑ}_{{𝔱}_{\theta \varphi }}^{\kappa },T_{\theta \varphi }^{\kappa }\right),\left({ℑ}_{{𝔩}_{\theta \varphi }}^{\kappa },L_{\theta \varphi }^{\kappa }\right)\right)$ to weigh his/her judgment on alternative ℧_θ_(θ = 1, 2, …, 6) over the attribute 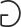
_φ_(φ = 1, 2, 3, 4).
**Step 2**. Establish the 2TL*T*-SF evaluation matrix as:
$$X={\left[{ℸ}_{\theta \varphi }^{\kappa }\right]}_{6\times 4}={\left(\left({ℑ}_{{𝔯}_{\theta \varphi }}^{\kappa },R_{\theta \varphi }^{\kappa }\right),\left({ℑ}_{{𝔱}_{\theta \varphi }}^{\kappa },T_{\theta \varphi }^{\kappa }\right),\left({ℑ}_{{𝔩}_{\theta \varphi }}^{\kappa },L_{\theta \varphi }^{\kappa }\right)\right)}_{6\times 4}$$
  where ${ℸ}_{\theta \varphi }^{\kappa }=\left(\left({ℑ}_{{𝔯}_{\theta \varphi }}^{\kappa },R_{\theta \varphi }^{\kappa }\right),\left({ℑ}_{{𝔱}_{\theta \varphi }}^{\kappa },T_{\theta \varphi }^{\kappa }\right),\left({ℑ}_{{𝔩}_{\theta \varphi }}^{\kappa },L_{\theta \varphi }^{\kappa }\right)\right)(\theta =1,2,\ldots ,6,\varphi =1,2,3,4,$ and κ = 1, 2, 3, 4) describes the 2TL*T*-SF data pertaining to options ℧_θ_ across attributes 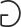
_φ_ from the perspective of DMs Π_κ_.
**Step 3**. Normalize the initial decision matrix:
  Given that all attributes are of beneficial types, it is unnecessary to normalize the preliminary decision matrix.
**Step 4**. Obtain the aggregated 2TL*T*-SF decision matrix according to the 2TL*T*-SFSSWA operator, the results are shown below Table 2:
**Step 5**. The score function matrix $\left({\overset{⌣}{\Omega }}_{\theta \varphi }\right)$ of the fused decision matrix is calculated by Equation 2 and then the obtained results are normalized.
  The results of score function matrix are given below:
$${\overset{⌣}{\Omega }}_{\theta \varphi }=\left[\begin{array}{ccccc} G_{1} & G_{2} & G_{3} & G_{4}\\ {℧}_{1} & 0.5447 & 0.5634 & 0.4804 & 0.4953\\ {℧}_{2} & 0.5023 & 0.6066 & 0.4948 & 0.4928\\ {℧}_{3} & 0.6014 & 0.5530 & 0.5172 & 0.4733\\ {℧}_{4} & 0.6655 & 0.5279 & 0.5081 & 0.6457\\ {℧}_{5} & 0.5279 & 0.4336 & 0.4810 & 0.4955\\ {℧}_{6} & 0.2770 & 0.5189 & 0.6031 & 0.4813 \end{array}\right]$$
  The results of normalized matrix are given below:
$${\bar{\Omega }}_{\theta \varphi }=\left[\begin{array}{ccccc} G_{1} & G_{2} & G_{3} & G_{4}\\ {℧}_{1} & 0.8185 & 0.9288 & 0.7965 & 0.7670\\ {℧}_{2} & 0.7548 & 1.0000 & 0.8204 & 0.7632\\ {℧}_{3} & 0.9037 & 0.9115 & 0.8577 & 0.7329\\ {℧}_{4} & 1.0000 & 0.8701 & 0.8425 & 1.0000\\ {℧}_{5} & 0.7932 & 0.7147 & 0.7976 & 0.7674\\ {℧}_{6} & 0.4162 & 0.8553 & 1.0000 & 0.7454 \end{array}\right]$$
**Step 6**. Equation 12 is used to compute ${♯}_{\theta }^{\left(1\right)}$, the initial quantity for the overall relative relevance, calculated with the weighted sum model. The obtained results are shown below:
**Step 7**. Equation 13 is used to compute ${♯}_{\theta }^{\left(2\right)}$, the subsequent overall relative relevance quantity, determined with the help of the weighted product model. The obtained results are shown below:
**Step 8**. ♯_θ_ represents the composite optimality value computed using Equation 14. χ denotes the coefficient for composite optimality, and it falls within the range of [0, 1]. When both the weighted sum model and the weighted product model equally influence the composite optimality criteria, χ assumes a value of 0.2. The combined optimality values are given below Table 3:
**Step 9**. Each alternative is ranked based on its ♯_θ_. The top-ranked alternative is the one that boasts the highest ♯_θ_ value.
  The ranking of alternatives is as follows:
$${℧}_{4}>{℧}_{3}>{℧}_{2}>{℧}_{1}>{℧}_{5}>{℧}_{6}$$
  Hence, Romycin is the best medication for the treatment of pink eye infection.

**Table 2:** Aggregated 2TL*T*-SF decision matrix.

	** 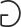 _1_**	** 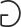 _2_**
℧_1_	((ℑ_6_, −0.3462), (ℑ_1_, 0.0576), (ℑ_5_, −0.4210))	((ℑ_6_, 0.3380), (ℑ_1_, 0.0971), (ℑ_6_, −0.3782))
℧_2_	((ℑ_4_, 0.4509), (ℑ_1_, 0.0619), (ℑ_4_, 0.3262))	((ℑ_6_, 0.3431), (ℑ_1_, 0.1042), (ℑ_5_, −0.4210))
℧_3_	((ℑ_6_, 0.2905), (ℑ_1_, 0.1042), (ℑ_5_, −0.4580))	((ℑ_6_, −0.2769), (ℑ_1_, 0.1042), (ℑ_4_, 0.4118))
℧_4_	((ℑ_7_, −0.2297), (ℑ_1_, 0.0388), (ℑ_5_, −0.3960))	((ℑ_6_, −0.3197), (ℑ_1_, 0.1042), (ℑ_5_, 0.1651))
℧_5_	((ℑ_5_, −0.0459), (ℑ_1_, 0.0576), (ℑ_2_, 0.3050))	((ℑ_5_, 0.2124), (ℑ_1_, 0.1042), (ℑ_6_, 0.1644))
℧_6_	((ℑ_3_, 0.0000), (ℑ_1_, 0.0307), (ℑ_7_, 0.0000))	((ℑ_5_, 0.0000), (ℑ_2_, 0.0341), (ℑ_4_, 0.2300))
	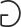 _3_	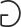 _4_
℧_1_	((ℑ_5_, 0.3438), (ℑ_2_, 0.0335), (ℑ_6_, −0.3200))	((ℑ_4_, 0.3643), (ℑ_1_, 0.0172), (ℑ_5_, −0.4063))
℧_2_	((ℑ_4_, 0.2995), (ℑ_1_, 0.1042), (ℑ_5_, −0.4348))	((ℑ_4_, 0.2341), (ℑ_2_, 0.0614), (ℑ_5_, −0.3963))
℧_3_	((ℑ_6_, −0.0850), (ℑ_1_, 0.0307), (ℑ_6_, −0.3143))	((ℑ_4_, −0.1846), (ℑ_1_, 0.0971), (ℑ_5_, 0.0753))
℧_4_	((ℑ_5_, −0.2823), (ℑ_2_, 0.1229), (ℑ_4_, 0.3517))	((ℑ_7_, −0.3910), (ℑ_1_, 0.0619), (ℑ_4_, 0.3656))
℧_5_	((ℑ_5_, −0.2438), (ℑ_1_, 0.0971), (ℑ_5_, 0.2745))	((ℑ_4_, −0.1268), (ℑ_1_, 0.0871), (ℑ_4_, 0.2294))
℧_6_	((ℑ_7_, −0.0974), (ℑ_1_, 0.0169), (ℑ_6_, 0.1501))	((ℑ_4_, 0.4230), (ℑ_2_, 0.1927), (ℑ_5_, 0.0842))

**Table 3:** Results of steps 6,7, and 8.

	**θ = 1**	**θ = 2**	**θ = 3**	**θ = 4**	**θ = 5**	**θ = 6**
${℧}_{\theta }^{\left(1\right)}$	0.8261	0.8314	0.8495	0.9317	0.7685	0.7465
${℧}_{\theta }^{\left(2\right)}$	0.0018	0.0018	0.0020	0.0028	0.0013	0.0010
	℧_1_	℧_2_	℧_3_	℧_4_	℧_5_	℧_6_
	0.2491	0.2507	0.2563	0.2815	0.2315	0.2247

### 6.3 Parameter analysis and discussion

As we know, the parameter vectors significantly influences the result of the decision. In this subsection, we look at how the parameters effect the combined optimality results and the final ranking results. As a result, investigating the impact of parameters k and *q* on decision results is critical. To do so, we make k and *q* a fixed sets and explore the impact of the results on ranking. The combined optimality values and rank correlation of options are organized in Tables 4, 5 considering various parameter choices of k and *q*. It is simple to conclude from it that the advantage of the SS operations is that they can render the decision-making process highly adaptable. The parameter can be thought of as a decision specialist's affection for uncertainty.

**Table 4:** Parameter analysis utilizing the parameter k by the 2TL*T*-SFSSWA.

**Parameters**	♯_**θ**_ **values**			**Ranking**
k = −25	♯_1_ = 0.2474	♯_2_ = 0.2389	♯_3_ = 0.2613	℧_4_ > ℧_3_ > ℧_1_ >
	♯_4_ = 0.2696	♯_5_ = 0.2183	♯_6_ = 0.2054	℧_2_ > ℧_5_ > ℧_6_
k = −20	♯_1_ = 0.2473	♯_2_ = 0.2394	♯_3_ = 0.2613	℧_4_ > ℧_3_ > ℧_1_ >
	♯_4_ = 0.2701	♯_5_ = 0.2181	♯_6_ = 0.2063	℧_2_ > ℧_5_ > ℧_6_
k = −17	♯_1_ = 0.2474	♯_2_ = 0.2399	♯_3_ = 0.2614	℧_4_ > ℧_3_ > ℧_1_ >
	♯_4_ = 0.2706	♯_5_ = 0.2181	♯_6_ = 0.2071	℧_2_ > ℧_5_ > ℧_6_
k = −15	♯_1_ = 0.2476	♯_2_ = 0.2403	♯_3_ = 0.2614	℧_4_ > ℧_3_ > ℧_1_ >
	♯_4_ = 0.2711	♯_5_ = 0.2183	♯_6_ = 0.2079	℧_2_ > ℧_5_ > ℧_6_
k = −13	♯_1_ = 0.2478	♯_2_ = 0.2410	♯_3_ = 0.2615	℧_4_ > ℧_3_ > ℧_1_ >
	♯_4_ = 0.2717	♯_5_ = 0.2187	♯_6_ = 0.2089	℧_2_ > ℧_5_ > ℧_6_
k = −12	♯_1_ = 0.2480	♯_2_ = 0.2414	♯_3_ = 0.2616	℧_4_ > ℧_3_ > ℧_1_ >
	♯_4_ = 0.2721	♯_5_ = 0.2190	♯_6_ = 0.2095	℧_2_ > ℧_5_ > ℧_6_
k = −11	♯_1_ = 0.2483	♯_2_ = 0.2420	♯_3_ = 0.2617	℧_4_ > ℧_3_ > ℧_1_ >
	♯_4_ = 0.2727	♯_5_ = 0.2194	♯_6_ = 0.2103	℧_2_ > ℧_5_ > ℧_6_
k = −9	♯_1_ = 0.2492	♯_2_ = 0.2435	♯_3_ = 0.2620	℧_4_ > ℧_3_ > ℧_1_ >
	♯_4_ = 0.2741	♯_5_ = 0.2208	♯_6_ = 0.2125	℧_2_ > ℧_5_ > ℧_6_
k = −7	♯_1_ = 0.2498	♯_2_ = 0.2449	♯_3_ = 0.2614	℧_4_ > ℧_3_ > ℧_1_ >
	♯_4_ = 0.2756	♯_5_ = 0.2224	♯_6_ = 0.2147	℧_2_ > ℧_5_ > ℧_6_
k = −3	♯_1_ = 0.2500	♯_2_ = 0.2490	♯_3_ = 0.2574	℧_4_ > ℧_3_ > ℧_1_ >
	♯_4_ = 0.2797	♯_5_ = 0.2285	♯_6_ = 0.2215	℧_2_ > ℧_5_ > ℧_6_
k = −1	♯_1_ = 0.2466	♯_2_ = 0.2520	♯_3_ = 0.2555	℧_4_ > ℧_3_ > ℧_2_ >
	♯_4_ = 0.2840	♯_5_ = 0.2367	♯_6_ = 0.2296	℧_1_ > ℧_5_ > ℧_6_
k = −0.7	♯_1_ = 0.2457	♯_2_ = 0.2521	♯_3_ = 0.2559	℧_4_ > ℧_3_ > ℧_2_ >
	♯_4_ = 0.2851	♯_5_ = 0.2394	♯_6_ = 0.2318	℧_1_ > ℧_5_ > ℧_6_

**Table 5:** Parameter analysis utilizing the parameter *q* by the 2TL*T*-SFSSWA.

**Parameters**	♯_**θ**_ **values**			**Ranking**
*q* = 25	♯_1_ = 0.2975	♯_2_ = 0.2974	♯_3_ = 0.2980	℧_4_ > ℧_3_ > ℧_1_ >
	♯_4_ = 0.2998	♯_5_ = 0.2965	♯_6_ = 0.2958	℧_2_ > ℧_5_ > ℧_6_
*q* = 20	♯_1_ = 0.2929	♯_2_ = 0.2925	♯_3_ = 0.2937	℧_4_ > ℧_3_ > ℧_1_ >
	♯_4_ = 0.2973	♯_5_ = 0.2907	♯_6_ = 0.2893	℧_2_ > ℧_5_ > ℧_6_
*q* = 17	♯_1_ = 0.2885	♯_2_ = 0.2879	♯_3_ = 0.2897	℧_4_ > ℧_3_ > ℧_1_ >
	♯_4_ = 0.2951	♯_5_ = 0.2849	♯_6_ = 0.2829	℧_2_ > ℧_5_ > ℧_6_
*q* = 15	♯_1_ = 0.2846	♯_2_ = 0.2838	♯_3_ = 0.2862	℧_4_ > ℧_3_ > ℧_1_ >
	♯_4_ = 0.2932	♯_5_ = 0.2795	♯_6_ = 0.2770	℧_2_ > ℧_5_ > ℧_6_
*q* = 13	♯_1_ = 0.2798	♯_2_ = 0.2787	♯_3_ = 0.2818	℧_4_ > ℧_3_ > ℧_1_ >
	♯_4_ = 0.2910	♯_5_ = 0.2726	♯_6_ = 0.2694	℧_2_ > ℧_5_ > ℧_6_
*q* = 12	♯_1_ = 0.2769	♯_2_ = 0.2756	♯_3_ = 0.2792	℧_4_ > ℧_3_ > ℧_1_ >
	♯_4_ = 0.2898	♯_5_ = 0.2684	♯_6_ = 0.2684	℧_2_ > ℧_5_ > ℧_6_
*q* = 11	♯_1_ = 0.2737	♯_2_ = 0.2723	♯_3_ = 0.2763	℧_4_ > ℧_3_ > ℧_1_ >
	♯_4_ = 0.2885	♯_5_ = 0.2636	♯_6_ = 0.2596	℧_2_ > ℧_5_ > ℧_6_
*q* = 9	♯_1_ = 0.2657	♯_2_ = 0.2646	♯_3_ = 0.2694	℧_4_ > ℧_3_ > ℧_1_ >
	♯_4_ = 0.2856	♯_5_ = 0.2521	♯_6_ = 0.2473	℧_2_ > ℧_5_ > ℧_6_
*q* = 7	♯_1_ = 0.2553	♯_2_ = 0.2556	♯_3_ = 0.2610	℧_4_ > ℧_3_ > ℧_2_ >
	♯_4_ = 0.2828	♯_5_ = 0.2385	♯_6_ = 0.2326	℧_1_ > ℧_5_ > ℧_6_
*q* = 3	♯_1_ = 0.2313	♯_2_ = 0.2353	♯_3_ = 0.2467	℧_4_ > ℧_3_ > ℧_2_ >
	♯_4_ = 0.2813	♯_5_ = 0.2236	♯_6_ = 0.2067	℧_1_ > ℧_5_ > ℧_6_
*q* = 1	♯_1_ = 0.2495	♯_2_ = 0.2459	♯_3_ = 0.2644	℧_4_ > ℧_3_ > ℧_5_ >
	♯_4_ = 0.2893	♯_5_ = 0.2524	♯_6_ = 0.2293	℧_1_ > ℧_2_ > ℧_6_
*q* = −0.7	♯_1_ = 0.2600	♯_2_ = 0.2558	♯_3_ = 0.2719	℧_4_ > ℧_3_ > ℧_5_ >
	♯_4_ = 0.2919	♯_5_ = 0.2614	♯_6_ = 0.2425	℧_1_ > ℧_2_ > ℧_6_

**(1) Parameter analysis with parameter** **k**

First, the parameter analysis is done with different parametric values of k(k = −25, −20, −17, −15, −13, −12, −11, −9, −7, −3, −1, −0.7) by fixing *q* = 6 and the results are depicted in Table 4. A judgment specialist's devotion to ambiguity might be contrasted to the parameter k. For all values of parameter k the best alternative is the same which is ℧_4_(℧_4_ = Romycin). Hence, Romycin is the best medication for the treatment of pink eye infection. The same ranking preference of alternatives shows the efficiency and accuracy of our proposed method. The choice inclination in the real MAGDM mechanism can be indicated by adjusting the settings of the parameter k to achieve the finest judgment conclusion.

**(2) Parameter analysis with parameter** **q**

Second, the parameter analysis is done with different parametric values of *q*(*q* = 25, 20, 17, 15, 13, 12, 11, 9, 7, 3, 1, 0.7) by fixing k = −2 and the results are depicted in Table 5. A judgment specialist's devotion to ambiguity might be contrasted to the parameter *q*. For all values of parameter *q* the best alternative is the same which is ℧_4_(℧_4_ = Romycin). Hence, Romycin is the best medication for the treatment of pink eye infection. The same ranking preference of alternatives shows the efficiency and accuracy of our proposed method. The choice inclination in the real MAGDM mechanism can be indicated by adjusting the settings of the parameter k to achieve the finest judgment conclusion.

In the final analysis, it is possible to discover that the sorting findings of variants are substantially varied regardless of how the parameters *q* and k are changed, which demonstrates that there is coherence in selecting the most suitable option. Though DMs alter the parameters *q* and k, most of the time, they will cling to the appropriate selections that were made during the decision-making process. This is a positive sign since it indicates that the choice-making process being offered is quite robust and comes to sound conclusions. It indicates that the process by which judgments are made is solid and dependable.

### 6.4 Comparative analysis and discussion

The link between options, the coherence of the attributes, and the objective assessments of the DMs all impact the choice of a MAGDM technique. We contrast the findings from the ranking with those attained in other publications to further support the soundness of the novel presented technique. Below is a summary of all the ranking findings that are produced using various techniques. In light of these findings, it is important to underline the subsequent points:

First, we can see that there are three different ranking findings that are obtained using diverse MAGDM techniques, and the variance between these findings is not large. Actually, according to practically all rankings, ℧_4_(℧_4_ = Romycin) is the best medication and ℧_1_(℧_1_ = Bleph-10) is the least. Second, take into account that the ranking findings gained through the suggested methodology deviate in comparison to those derived from the 2TL*T*-SF-CODAS method (Akram et al., 2023a), the 2TL*T*-SF-MABAC method (Akram et al., 2023b), the 2TL*T*-SF-EDAS method (Naz et al., 2022) in general.

**(1) Comparative analysis with the 2TLT-SF-CODAS method (Akram et al.**, **2023a****)**.

The WASPAS and CODAS methods are both used to address issues in MAGDM. To address issues with MAGDM, the WASPAS and CODAS approaches offer the following features:

1. Decision matrix: WASPAS uses a decision matrix that consists of alternatives and their corresponding attributes and attribute weights. CODAS utilizes a decision matrix where alternatives are compared to a pre-defined ideal solution.
2. Aggregation technique: WASPAS combines the sum and product of attribute values, incorporating both additive and multiplicative aggregation. CODAS employs complex proportional assessment, considering the closeness of each alternative to the ideal solution.
3. Weighting: Weighting is a crucial aspect in WASPAS, allowing DMs to assign different levels of importance to each attribute. CODAS does not involve explicit weighting; instead, it ranks alternatives based on their proximity to the ideal solution.
4. Ideal and anti-ideal solutions: WASPAS usually includes ideal and anti-ideal solutions for each attribute to calculate the distance of each alternative from these reference points. CODAS employs a fixed ideal solution against which alternatives are compared to determine their relative desirability.
5. Applicability: WASPAS is well-suited for scenarios where DMs have a clear understanding of attribute importance and trade-offs. CODAS is useful in situations where the ideal solution is predefined, and it is important to assess how well alternatives match this ideal.
6. Sensitivity analysis: WASPAS can be sensitive to the choice of weights, making sensitivity analysis an essential step in the decision-making process. CODAS is less sensitive to weight variations due to its proportional assessment approach.
7. Output: WASPAS is a ranking or score for each alternative, indicating their overall desirability. CODAS provides a ranking of alternatives based on their proximity to the ideal solution.
8. Complexity: WASPAS can be more complex to implement, especially when dealing with a large number of attributes and alternatives. CODAS is relatively simpler and more intuitive in terms of methodological complexity.

Both WASPAS and CODAS offer valuable tools for addressing MAGDM issues, and the choice between them depends on the specific requirements and characteristics of the decision-making problem at hand. The ranking findings of the proposed 2TL*T*-SF-WASPAS approach and the existing 2TL*T*-SF-CODAS approach (see Table 6) reveals that the best alternative is different which demonstrate the proficiency of our proposed approach.

**Table 6:** Ranking of the alternatives utilizing 2TL*T*-SF-WASPAS and 2TL*T*-SF-CODAS approaches.

**MAGDM methods**	**Ranking**
2TL*T*-SF-WASPAS	℧_4_ > ℧_3_ > ℧_2_ > ℧_1_ > ℧_5_ > ℧_6_
2TL*T*-SF-CODAS	℧_2_ > ℧_5_ > ℧_4_ > ℧_3_ > ℧_6_ > ℧_1_

**(2) Comparative analysis with the 2TLT-SF-MABAC method (Akram et al.**, **2023b****)**.

The WASPAS and MABAC methods are both powerful tools for addressing MAGDM issues. To address issues with MAGDM, the WASPAS and MABAC approaches offer the following features:

1. Scalability: Both WASPAS and MABAC are scalable and can handle a large number of attributes and criteria, making them suitable for complex MAGDM scenarios.
2. Aggregation techniques: WASPAS focuses on weighted aggregation, allowing DMs to assign different weights to attributes based on their importance. MABAC, on the other hand, uses border approximation for aggregation, providing a different perspective on the attribute values.
3. Decision maker involvement: Both methods take into account the preferences and involvement of DMs. WASPAS enables the use of weighted sum and weighted product methods to reflect the preferences of DMs, while MABAC allows for assessing the boundary values that define decision areas.
4. Objective and subjective information: WASPAS and MABAC can handle both objective and subjective information. DMs can provide subjective evaluations and preferences while incorporating objective data into the decision-making process.
5. Flexibility: These methods are flexible in terms of incorporating various decision-making preferences, such as maximizing or minimizing objectives, which is essential in MAGDM where conflicting goals may exist.
6. Ranking and sorting: Both methods offer the capability to rank and sort alternatives based on their performance according to the defined criteria, facilitating a clear decision-making process.
7. Sensitivity analysis: Sensitivity analysis can be performed with both WASPAS and MABAC to assess how changes in the input data, criteria weights, or preferences affect the final decision outcomes, enhancing the robustness of the decision-making process.
8. Transparency: The methods provide transparency in the decision-making process by allowing DMs to see how the alternatives are evaluated and ranked, which is crucial for decision legitimacy and understanding.
9. Applicability: WASPAS and MABAC can be applied to a wide range of domains, including engineering, economics, environmental management, and more, making them versatile for various MAGDM scenarios.

By using the strengths of WASPAS and MABAC, DMs can benefit from a more comprehensive approach to address MAGDM issues, considering various aspects of their decision problem and gaining deeper insights into the alternatives' performance. The ranking findings of the proposed 2TL*T*-SF-WASPAS approach and the existing 2TL*T*-SF-MABAC approach (see Table 7) reveals that the best alternative is the same which demonstrate the efficiency of our proposed approach.

**Table 7:** Ranking of the alternatives utilizing 2TL*T*-SF-WASPAS and 2TL*T*-SF-MABAC approaches.

**MAGDM methods**	**Ranking**
2TL*T*-SF-WASPAS	℧_4_ > ℧_3_ > ℧_2_ > ℧_1_ > ℧_5_ > ℧_6_
2TL*T*-SF-MABAC	℧_4_ > ℧_5_ > ℧_2_ > ℧_6_ > ℧_3_ > ℧_1_

**(3) Comparative analysis with the 2TLT-SF-EDAS method (Naz et al.**, **2022****)**.

The WASPAS and EDAS methods are both techniques used to address MAGDM issues. To address issues with MAGDM, the WASPAS and EDAS approaches offer the following features:

1. Scalability: Both WASPAS and EDAS are scalable methods, capable of handling a wide range of attributes and alternatives. This makes them suitable for complex decision-making scenarios with numerous factors to consider.
2. Objective evaluation: These methods provide objective evaluations of alternatives based on a systematic and mathematical approach, reducing the subjectivity in decision-making processes.
3. Objective evaluation: These methods provide objective evaluations of alternatives based on a systematic and mathematical approach, reducing the subjectivity in decision-making processes.
4. Weighting of attributes: WASPAS assigns weights to attributes, reflecting their relative importance in the decision-making process. This ensures that the decision is influenced by the significance of each attribute.
5. Distance-based analysis: EDAS focuses on evaluating alternatives based on their distance from the average solution. This distance metric provides insights into how alternatives deviate from the central tendency, aiding in the identification of the most suitable options.
6. Sensitivity analysis: The usage of WASPAS and EDAS allows for sensitivity analysis, which helps in assessing the robustness of the decision by analyzing how changes in attribute weights or alternative values impact the final ranking.
7. Transparency: Both methods offer transparency in the decision-making process by providing clear and understandable results, enabling DMs to justify their choices and communicate the rationale behind their decisions.
8. Multi-criteria decision analysis: The integration of WASPAS and EDAS aligns with the principles of multi-criteria decision analysis, making it a comprehensive approach for addressing complex MAGDM problems where multiple criteria and stakeholders are involved.
9. Applicability: WASPAS and EDAS can be applied to a variety of domains, including business, engineering, environmental management, and healthcare, making them versatile tools for addressing MAGDM challenges in different contexts.

By combining the features of WASPAS and EDAS, DMs can enhance their ability to make informed, transparent, and collectively agreed-upon decisions in complex decision-making scenarios involving multiple attributes and alternatives. The ranking findings of the proposed 2TL*T*-SF-WASPAS approach and the existing 2TL*T*-SF-EDAS approach (see Table 8) reveals that the best alternative is the same which demonstrate the efficiency of our proposed approach.

**Table 8:** Ranking of the alternatives utilizing 2TL*T*-SF-WASPAS and 2TL*T*-SF-EDAS approaches.

**MAGDM methods**	**Ranking**
2TL*T*-SF-WASPAS	℧_4_ > ℧_3_ > ℧_2_ > ℧_1_ > ℧_5_ > ℧_6_
2TL*T*-SF-EDAS	℧_4_ > ℧_3_ > ℧_2_ > ℧_1_ > ℧_5_ > ℧_6_

**(4) Comparative analysis with the 2TLT-SF Entropy and maximizing deviation methods (Alamoodi et al.**, **2024****; Naz et al.**, **2023a****)**.

The WASPAS, Entropy, and maximizing deviation methods are techniques used to address MAGDM issues. To address issues with MAGDM, the WASPAS, Entropy, and maximizing deviation approaches offer the following features:

1. Multi-criteria evaluation: All three methods are used for evaluating alternatives across multiple criteria, enabling comprehensive analysis of complex decision-making problems. WASPAS integrates additive and multiplicative criteria, while Entropy and maximizing deviation methods handle criteria weighting and distinction between alternatives.
2. Objective weight calculation: The Entropy method provides an objective way to calculate criterion weights by analyzing the distribution of data across criteria. It assigns higher weights to criteria with higher variability, indicating greater importance based on information content.
3. Weight adjustments based on data variability: The maximizing deviation method aligns with Entropy by emphasizing the variance in data for each criterion. It maximizes the difference between criteria values, helping to refine the criteria weights based on how distinct the alternatives are from one another.
4. Hybrid scoring approach: WASPAS combines weighted sum and product models, allowing flexibility in decision analysis by leveraging both additive and multiplicative aggregation. This enables better adaptability to various types of data and criteria relationships.
5. Incorporation of both subjective and objective weights: By combining WASPAS and Entropy with maximizing deviation, DMs can incorporate both subjective preferences and objective weight adjustments, ensuring a more balanced evaluation of alternatives.
6. Sensitivity to information distribution: Entropy is sensitive to how information is distributed across alternatives for each criterion, helping to distinguish between criteria that offer unique information. This makes it valuable in highlighting key differentiators in decision-making.
7. Enhanced decision accuracy: By integrating the strengths of WASPAS with Entropy and maximizing deviation, the combined approach enhances accuracy, robustness, and consistency in decision-making.

By combining the features of WASPAS with Entropy and maximizing deviation, DMs can enhance their ability to make informed, transparent, and collectively agreed-upon decisions in complex decision-making scenarios involving multiple attributes and alternatives. The ranking findings of the proposed 2TL*T*-SF-WASPAS approach and the existing 2TL*T*-SF-Entropy approach (see Table 9) reveals that the best alternative is the same but the best alternative is different for the existing 2TL*T*-SF maximizing deviation method which demonstrate the efficiency of our proposed approach.

**Table 9:** Ranking of the alternatives utilizing 2TL*T*-SF Entropy and maximizing deviation approaches.

**MAGDM methods**	**Ranking**
2TL*T*-SF-WASPAS	℧_4_ > ℧_3_ > ℧_2_ > ℧_1_ > ℧_5_ > ℧_6_
2TL*T*-SF-Entropy	℧_4_ > ℧_3_ > ℧_2_ > ℧_1_ > ℧_6_ > ℧_5_
2TL*T*-SF maximizing deviation	℧_4_ > ℧_3_ > ℧_1_ > ℧_2_ > ℧_5_ > ℧_6_

## 7 Conclusions

The SSWA and SSWG AOs are recognized for their capacity to handle difficult and unreliable data since they are generalized to a far greater extent than many other AOs. The WASPAS technique is best recognized for its simplicity to rank the alternatives. Truth, abstinence, falsity, and 2TL information are the four key terms that make up the 2TL*T*-SFS, the overall structure under consideration exhibits a comprehensive set of characteristics, making it a highly strong notion. Furthermore, it is a very difficult challenge for researchers to aggregate the collection of 2TL*T*-SF data into singleton 2TL*T*-SF data. So, for the mentioned problem, we offer the following important and dominant solutions in this manuscript.

1. In consideration of the concepts of *t*-norm and *t*-conorm, we have elaborated on the 2TL*T*-SFS and its associated operational principles.
2. We investigated the 2TL*T*-SFSSWA and 2TL*T*-SFSSWG operators, which are a combined version of the 2TL*T*-SFS, SSWA, and SSWG AOs.
3. The essential attributes of the previously mentioned details, namely idempotency, monotonicity, and boundedness, were inferred.
4. In this study, we employed a MAGDM technique known as 2TL*T*-SF-WASPAS to effectively determine the optimal drug for the treatment of pink eye infection.
5. In order to demonstrate the superiority of the suggested strategy, we conducted a parameter analysis and comparison analysis of the aforementioned strategy, taking into account several current shortcomings, within the context of a realistic situation.

The proposed research has some limitations which are as follows: The 2TL*T*-SF information is a superior and valuable concept compared to an accumulation of FS theories, but we also observed that the theory of the 2TL*T*-SFS needs serious modification once more because it failed when the notion of reality, abstention, and falsehood was employed, it was done so in the context of hesitant fuzzy numbers. Sometimes confusing information makes it harder to manage data. More specifically, some results are only applicable in certain circumstances. Due to the ambiguity and uncertainty in the fuzzy alternative's structure, quantitative measures become challenging. The subsequent enumeration outlines our prospective objectives for study: We explain how the derived 2TL*T*-SF information and their AOs will be applied in a variety of settings, involving fictitious intellect, predictive modeling, theoretical gaming, neurological connections, transportation indications, computational technology, feature acknowledgment, healthcare assessment, accumulating assessment, assessment of risks, and program design. We enhance knowledge for workload prediction, focusing on sophisticated computational platforms (Wang et al., 2022) as well as fractional-order developing knowledge (Mohammadzadeh et al., 2019).

## Data Availability

The original contributions presented in the study are included in the article/supplementary material, further inquiries can be directed to the corresponding author.
